# The Development of Pharmacophore Models for the Search of New Natural Inhibitors of SARS-CoV-2 Spike RBD–ACE2 Binding Interface

**DOI:** 10.3390/molecules27248938

**Published:** 2022-12-15

**Authors:** Valentin A. Semenov, Leonid B. Krivdin

**Affiliations:** A. E. Favorsky Irkutsk Institute of Chemistry, Siberian Branch of the Russian Academy of Sciences, Favorsky St. 1, 664033 Irkutsk, Russia

**Keywords:** induced fit docking, natural compound, SARS-CoV-2 inhibitors, spike RBD, human ACE2, pharmacophore model

## Abstract

To date, some succeeding variants of SARS-CoV-2 have become more contagious. This virus is known to enter human cells by binding the receptor-binding domain (RBD) of spike protein with the angiotensin-converting enzyme 2 (ACE2), the latter being a membrane protein that regulates the renin–angiotensin system. Since the host cell receptor plays a critical role in viral entry, inhibition of the RBD–ACE2 complex is a promising strategy for preventing COVID-19 infection. In the present communication, we propose and utilize an approach based on the generation of a complex of pharmacophore models and subsequent Induced Fit Docking (IFD) to identify potential inhibitors of the main binding sites of the Omicron SARS-CoV-2 RBD(S1)–ACE2 complex (PDB ID: 7T9L) among a number of natural products of various types and origins. Several natural compounds have been found to provide a high affinity for the receptor of interest. It is expected that the present results will stimulate further research aimed at the development of specialized drugs against this virus.

## 1. Introduction

SARS-CoV-2 is a virus of the species (SARS-CoV), causing severe acute respiratory syndrome, and is related to the SARS-CoV-1 virus that caused the 2002–2004 SARS outbreak. Available evidence indicates that it is most likely of zoonotic origins and has close genetic similarity to bat coronaviruses, suggesting it emerged from a bat-borne virus. The virus shows little genetic diversity, indicating that the spillover event introducing SARS-CoV-2 to humans is likely to have occurred in late 2019. Epidemiological studies estimate that each infection resulted in an average of 2.4 to 3.4 new ones when no members of the community are immune and no preventive measures are taken. However, some subsequent variants have become more infectious. The virus primarily spreads between people through close contact and via aerosols and respiratory droplets that are exhaled when talking, breathing, or otherwise exhaling, as well as those produced from coughs or sneezes. It enters human cells by binding to ACE2, a membrane protein that regulates the renin–angiotensin system.

**Figure 1:**
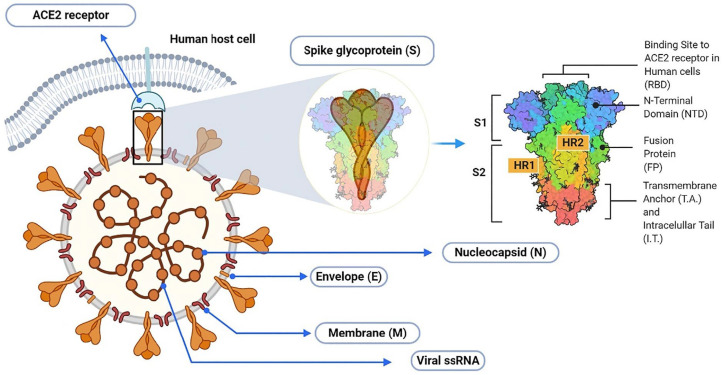
Schematic illustration of SARS-CoV-2 virion with detailed arrangement of spike receptor binding domain. Reproduced with minor editing privilege from Abubakar [9] under the Creative Commons Attribution License (CC BY).

It is well known that SARS-CoV-2 is an enveloped single-stranded RNA virus with the spike-shaped glycoproteins protruding from its outer surface of the membrane, thus forming a “crown” [1]. It has four main structural proteins: spike, envelope, membrane, and nucleocapsid. The spike protein, in turn, exists in a trimeric form, with each protomer having two functional subunits, S1 and S2, as shown in Figure 1. The S1 subunit includes the receptor-binding domain (RBD), which is responsible for the recognition of the angiotensin-converting enzyme 2 of the host cell and determines the range of potential carriers, which is an important step for the introduction of the virus core into the cell [2,3,4,5,6,7].

At this stage, the virus attaches to the cell surface, and the spike protein, in turn, is exposed to the host protease to initiate infection. This mechanism is shared by several known human pathogenic coronaviruses. At the same time, RBD, in the process of interaction with ACE2, makes hinged movements to move from the lower state to the upper one to remove steric hindrances [8].

Since the host cell receptor plays a critical role in viral entry, inhibition of the RBD–ACE2 complex is a promising strategy for preventing COVID-19 infection. However, it should be noted that, for a number of reasons, peptide inhibitors have some disadvantages that limit their use as drugs, in particular, poor metabolic stability, poor membrane permeability, and rapid clearance [10]. Taking this fact into account, the development of low-molecular-weight drugs that can either prevent the interaction of RBD with ACE-2 or affect the stability of an already formed complex is a very urgent task [11,12,13,14,15,16,17].

To date, two main strategies have been formed to prevent the penetration of viral RNA into the cell. The first strategy to inhibit viral attachment eliminates the binding of the spike protein to the ACE2 enzyme by monoclonal antibodies directed to the receptor-binding domain of the spike protein. The second strategy involves the distortion of the main RBD–ACE2 interaction interface due to its binding to blocking ligands or compounds that modify the glycan component of human ACE2. Of course, the implementation of the second strategy is possible both with the use of known structures that inhibit other viral proteins as well as with the use of completely new compounds that will have affinity for the RBD–ACE2 binding interface [18,19].

However, the search for the structures, which may be used for further drug development, is an extremely long and expensive process. From this point of view, the wide structural diversity and unique properties of natural products (NPs) predetermine them to be a good starting point, serving as a convenient template for the development of new inhibitors. Natural products with known antiviral activity may also be an additional means of fighting SARS-CoV-2 infection. At the moment, a number of theoretical works have been published that describe the binding abilities and dynamic behavior of many natural products relative to the RBD–ACE2 complex [9,20,21,22,23,24,25,26,27], as well as that considered the advantages in therapy, as compared to monoclonal antibodies [9,27,28,29,30,31].

It is reasonable to assume that most of them indicate the interruption of the interaction between RBD and ACE2 through competitive or allosteric inhibition by small-molecular-weight ligands [32,33,34,35,36]. Taking into account the experience of previous studies, we propose in this work to consider several binding domains, including those in the cleft and on the surface of ACE2. This approach will make it possible to identify not only potential inhibitors of the main contact zone but also the allosteric inhibitors of the formation of the RBD–ACE2 complex, which exhibits high complementarity to the ACE2 enzyme. To do this, we analyzed the binding affinity of about 25,000 compounds taken from a specialized database (DB) of natural products, COCONUT [37]. The library of potential natural ligands was directed to one of the latest SARS-CoV-2 cryo-EM structures SARS-CoV-2 Omicron RBD(S1) in complex with ACE2 (PDB ID: 7T9L [38]) for the virtual screening and subsequent extra-precision molecular docking.

As is well known, the development of new drugs is based not only on a classical docking of a ligand into the protein structure, but also on the search for regularities in the structure of the potential inhibitors. In this line, we propose in this study an in silico ligand search technique based on complex pharmacophore modeling. The developed 3D pharmacophore models make it possible to carry out the rapid virtual screening of a large DB of compounds. The pharmacophore model is a spatial set of steric and stereoelectronic features required by the receptor for the molecular recognition of a ligand. At the same time, one of its main advantages is manifested in the possibility of explaining how structurally different ligands are able to interact with a common binding domain. According to the literature, there are several examples of the use of pharmacophore models to search for new SARS-CoV-2 receptor ligands (not only the RBD–ACE2 complex), which have a predominantly quite local character [39,40,41,42,43].

Thus, within the framework of the present study, we have developed a set of pharmacophore hypotheses with the aim of coverage and the further screening of the structurally diverse potential natural inhibitors of the interaction of the SARS-CoV-2 spike protein with angiotensin-converting enzyme 2.

## 2. Results and Discussion

### 2.1. Initial Identification of Structural Similarity

**Figure 2:**
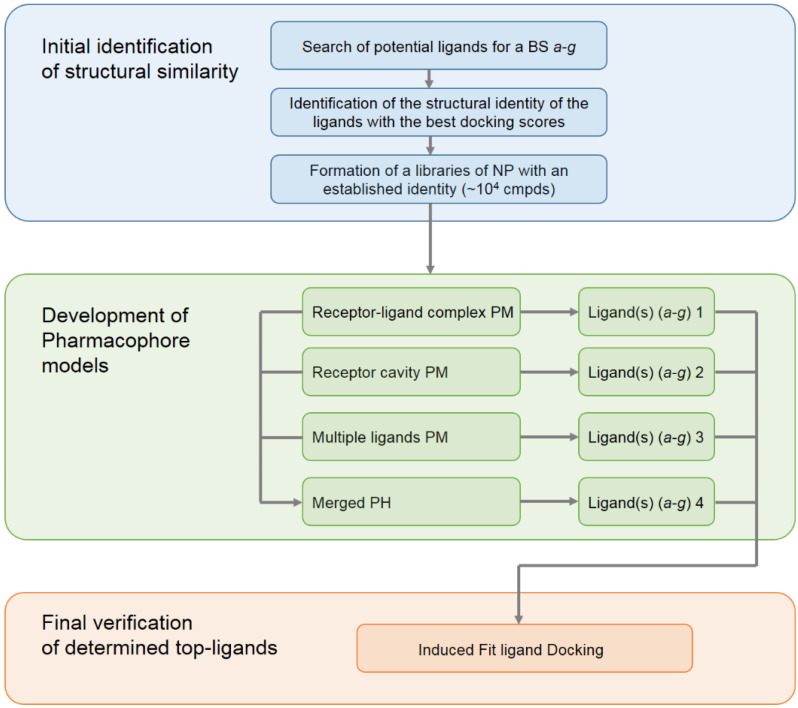
The general scheme of the workflow for the development of pharmacophore models used for the search of new inhibitors (carried out and applied in the present study).

As has already been mentioned, the selection and development of low-molecular-weight structures capable of disrupting the interaction of RBD with ACE2 is currently an urgent task. In this study, to search for new potential inhibitors of the SARS-CoV-2 spike RBD binding to ACE2, we propose an integrated approach based on the development of pharmacophore models and the subsequent screening of a database of NPs based on these models. This study consisted of three main blocks with the first one dealing with the initial identification of structural similarity; see Figure 2.

**Figure 3:**
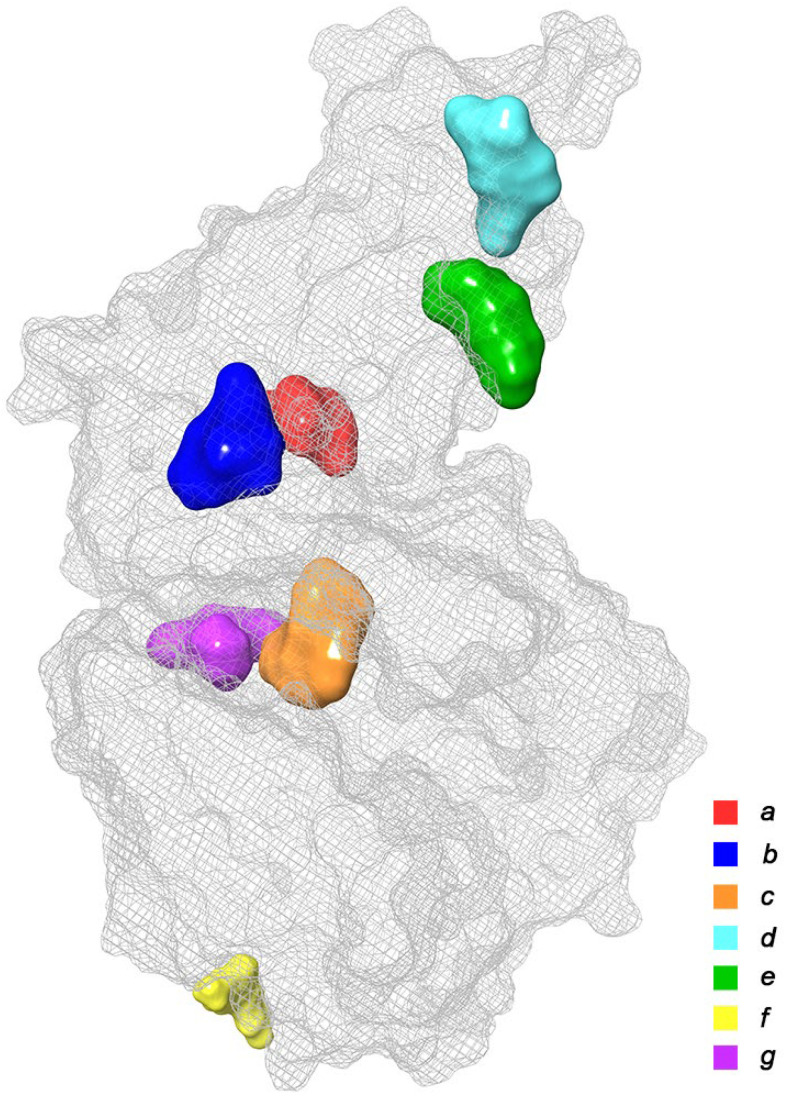
Main binding domains of potential ligands to receptors RBD и ACE2 (based on data provided in Refs. [20,21,22,23,24,25,26,27,28,29,30,31,32,33,34,35,36,37,38,39,40,41,42,43,44,45,46,47,48,49,50,51,52,53,54,55,56,57,58,59,60,61,62,63,64,65].

At this stage, we conducted a small literature review and tried to summarize the available data on the known in silico investigations of the main binding domains of potential ligands (both natural and synthetic) with the RBD and ACE2 receptors; see Figure 3. In total, about seven basic sites were defined, namely (*a*)—the RBD–ACE2 binding interface [20,21,22,23,29,40,44,45,46,47,48,49,50,51,52,53,54,55]; (*b*,*e*)—three pockets on the surface of the RBD protein near the central contact zone [21,26,46,56,57] and (*d*)—in the recess of a bended hydrophobic “tube” [24,58,59]; (*c*)—large catalytic cleft in the cavity of ACE2 [20,25,41,44,45,60,61,62]; (*f*)—surface binding site ACE2 [20,57]; and finally, (*g*)—a tight pocket in the core of ACE2 near its cleft [41,63,64,65]. The potential inhibitors of the listed base domains are presented in Table 1, while their residues are described in Table 2.

For each domain, characteristic sets of ligands with high binding energies with the receptor were selected according to the literature data. At this stage, we deliberately did not give preference to natural ligands, which was in view of the fact that we needed to determine the generality, as well as the similarity of the structure that exhibits high affinity for a specific binding site. Also, at this stage we did not filter out the structures that have reactive functional groups, being thus capable of high chemical reactivity.

At the next stage, these sets of selected ligands were prepared for molecular docking (see Section 3.4.), and their ground tautomeric states were generated. After docking, the ligands were differentiated by their binding energies. In each of the sets *a*–*g*, some 1–3 compounds possessing top docking scores were established. For those compounds, the similarity of their structural fragments was determined. Based on this similarity, a search for natural products in the COCONUT DB was performed. In order to cover as many potential inhibitors as possible, we set the Tanimoto similarity [85] threshold to 85–95%. Thus, for each of the domains *a*–*g*, libraries were formed from as many as about 1000–5000 natural products possessing a certain structural similarity.

### 2.2. Development of Pharmacophore Models

At the next main stage of this study, pharmacophore models were developed that reflect the specificity of interactions in the considered binding pocket. For each of the considered domains *a*–*g*, as many as four pharmacophore models were constructed (28 in total). Herein, we will consider these models on an example of pocket *b*. The graphic representation and spatial arrangement of all four models for set *b* are represented in Figure 4, Figure 5, Figure 6 and Figure 7 while the remaining structures of the developed pharmacophore models are given in the Supplementary Materials; see Figures S1–S24.

**Figure 4:**
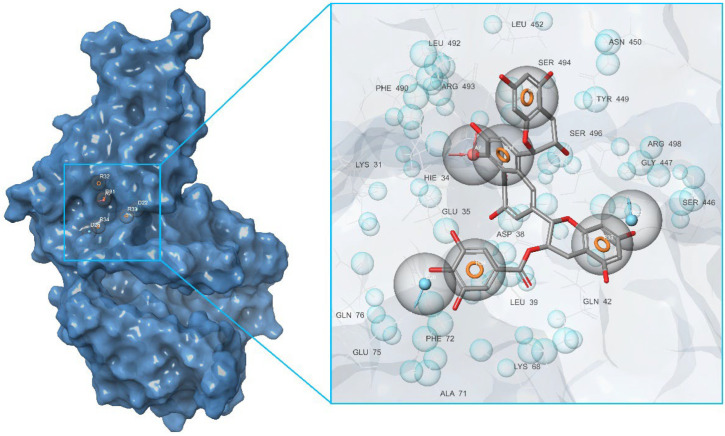
Pharmacophore model of the RBD site *b* of spike protein generated by the Receptor-ligand method.

First of all, based on the results of the molecular docking performed at the previous stage, the lowest energy complex of the receptor with ligand from set *b* was determined. Based on the analysis of chemical interactions of this complex, the “Receptor-ligand” hypothesis was generated. The structure of the resulting hypothesis is given in Table 3. It follows that acceptor A7 lies in the vicinity of His34 and D22 is located near Ser446, while D26 is near Gln76 (see Figure 4). Aromatic rings R32, R33, and R34 are located near Ser494, Gln42, and Asp38, respectively.

**Figure 5:**
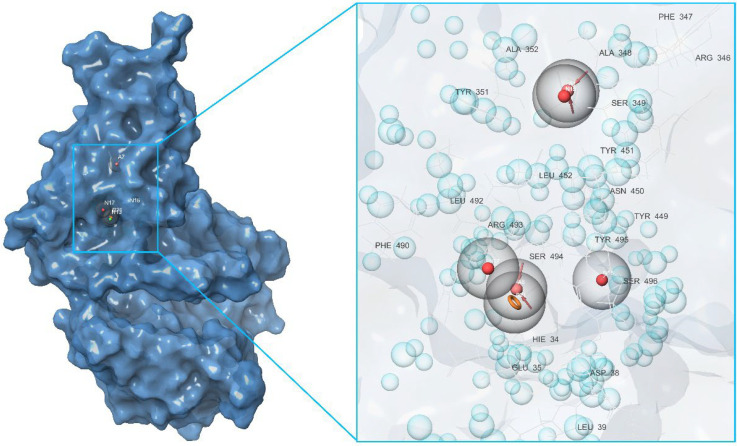
Pharmacophore model of the RBD site *b* of a spike protein generated by the Receptor cavity method.

**Figure 6:**
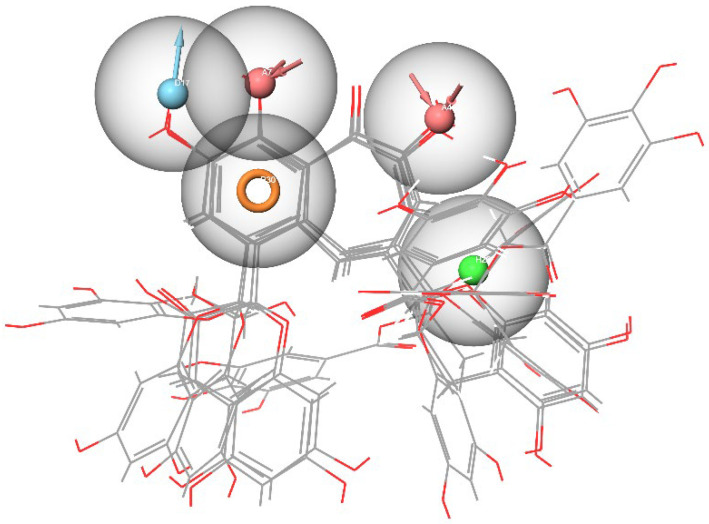
Pharmacophore model of the RBD site *b* of the spike protein generated by the Multiple ligands method.

The second model was derived from the cavity of the corresponding receptor binding site. Herein, we can also observe that the dyad of the aromatic ring and A8 acceptor is located in the cavity of Arg493-Ser494-His-34, as shown in Figure 5. Based on this fact, one can draw a conclusion about the structural commonality of the first and second models. Further, as part of the construction of the “Multiple ligands” hypothesis, a complex of three models was generated based on the entire set of ligands *b*, as presented in Figure 6. It can be seen that their structures contain two acceptors based on the hydroxyl groups of the benzoannulenone moiety, at least one aromatic ring, and a hydrophobic center.

**Figure 7:**
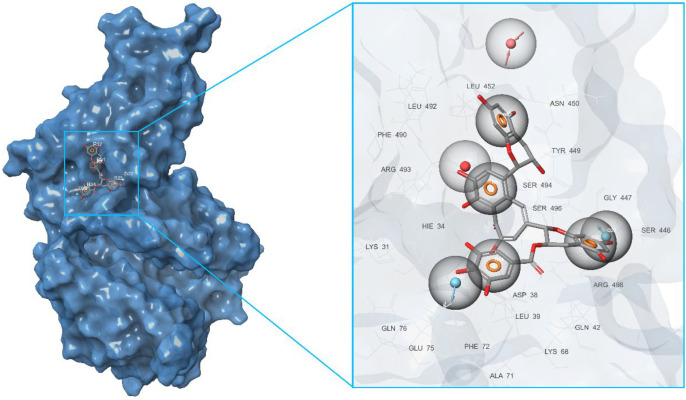
Pharmacophore model of the RBD site *b* of a spike protein generated by the Merged hypothesis method.

Furthermore, in the framework of this study, we tried to create a merged hypothesis that takes into account the structures of the three hypotheses presented above. As a result of the generation of the merged hypothesis, a model was obtained that includes the common features of the previous three models. It can be seen that, in this case, features, such as A1, D22, D36, N4, R31, R32, R33, and R34, were retained; see Figure 7. In general, here, as well as in the receptor-ligand and receptor cavity models, one can observe the arrangement of a number of aromatic cycles in the Arg493-Ser494-Arg498-His-34 cavity. This indicates the most preferred configuration of the potential ligand for a given binding site.

In order to determine the set of the key ligands for each of the developed pharmacophore models of all studied domains, a virtual screening was carried out using the libraries of NPs formed at the first stage; see the flow chart in Figure 2. Based on the developed hypothesis, the suitability of compounds for the corresponding pharmacophore model is analyzed during the screening process. By analyzing the mapping of screening ligands to the model structure, the Phase module ranks the virtual screening results based on the suitability score, known as the “Phase Screen Score”. The latter determines the complementarity of the ligand to the given pharmacophore model.

Another indicator, the “Matched Ligand Site”, indicates which particular features of the hypothesis turn out to be appropriate for the particular ligand under consideration. The corresponding natural products array screening parameters for the key ligands are provided in Table 3. Based on the proposed procedure, as many as seven key ligands were selected for the corresponding binding pocket from the library *b* consisting of 3632 compounds.

Analyzing the screening results within the Multiple ligands model of the *b* domain, one can note a clear predominance of the CNP0131499 compound in its group with a Phase Screen Score of about 1.898. Herewith, four features turned out to be appropriate for this ligand, namely A4, D17, H26, and R30. It should be noted that, in some cases, we expanded the number of key ligands for one model to 3–4 pieces, since their Phase Screen Scores turned out to be rather large and, moreover, very close among the compounds of their screening library. In this way, for each of the developed pharmacophore models 1–4 of all studied domains *a*–*g*, the key ligands were found that show complementarity to their binding sites.

### 2.3. Final Verification of Key Ligands

After the key ligands for all 28 models were found, we proceeded to their comprehensive testing in order to identify those with the highest inhibition potential. In order to take into account distortions in the protein structure together with its side chains during its interaction with the studied ligands, we used the Induced Fit Docking protocol, which plays a crucial role in the process of extra-precision docking [86]. The IFD approach makes it possible to take into account both the flexibility of the docked ligand as well as the flexibility of the receptor, while adjusting the spatial structure of the latter based on the docked ligand. In that way, all key ligands were subjected to the IFD protocol with the selected 3D structure of the spike RBD–ACE2 complex (PDB ID: 7T9L).

The results of that survey were analyzed based on the affinity of the docked ligands to their binding domains. It followed that a number of compounds, namely CNP0332318, CNP0401960, CNP0277806, CNP0380471, CNP0340958, CNP0393256, and CNP0125042 had a binding energy lower than the other key ligands for binding sites *a*–*g*, respectively. As a result, those compounds showed a good coordination with binding affinity, namely of −6.71, −7.93, −9.98, −8.68, −6.21, −6.56, and −9.16 kcal/mol, respectively.

**Figure 8:**
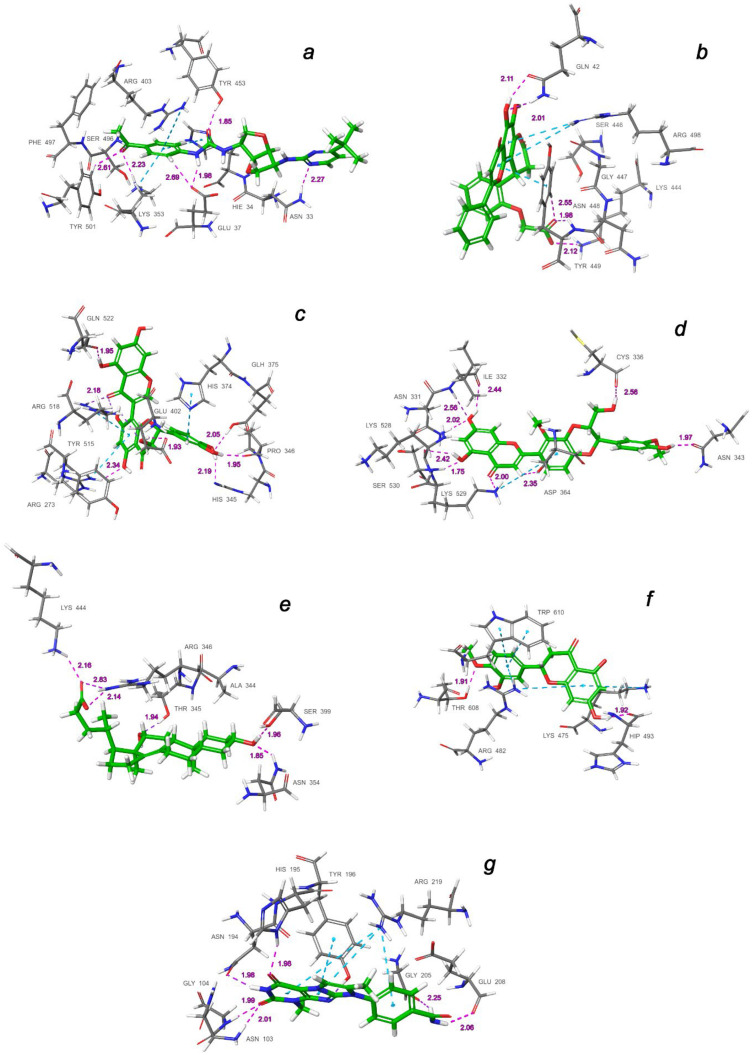
Interactions of the top-ligands at binding sites (**a**–**g**) of SARS-CoV-2 spike RBD–ACE2 (PDB ID: 7T9L). Hydrogen bond lengths with key residues are given in angstroms and shown as dashed purple lines. *π*–alkyl and *π*–stacking contacts are shown as dashed turquoise lines.

**Figure 9:**
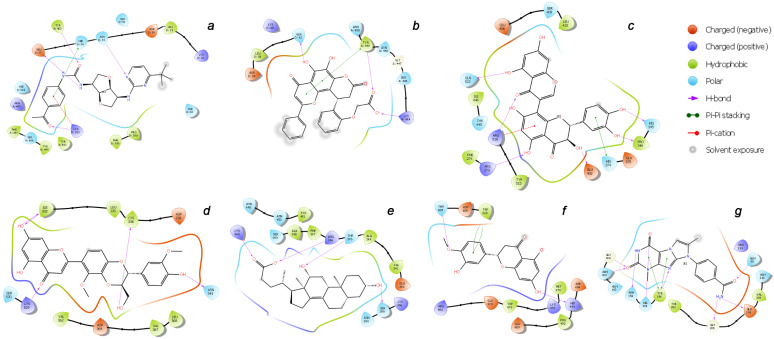
2D binding interaction diagrams of the top-ligands inside the (**a**–**g**) active sites of RBD–ACE2 (PDB ID: 7T9L).

It follows from the data presented in Table 3 that the best ligand in the IFD analysis is not always the one with the highest screening score. This indicates that the flexibility and variability of the protein structure in the region of the binding domain correct the resulting energy of interaction with the ligand during docking. Table 4 shows the final result of the performed IFD calculations in the most ranked binding position arranged in the order of decreasing predicted free binding energy, Δ*G*. The extensive interactions of the docking poses of the top-ligands with binding domains *a*–*g* are shown in Figure 8, while their corresponding 2D interaction diagrams are presented in Figure 9. These contacts include hydrogen bonds (HBs), van der Waals interactions, *π*-alkyl, and *π*–*π* stacking.

**Figure 10:**
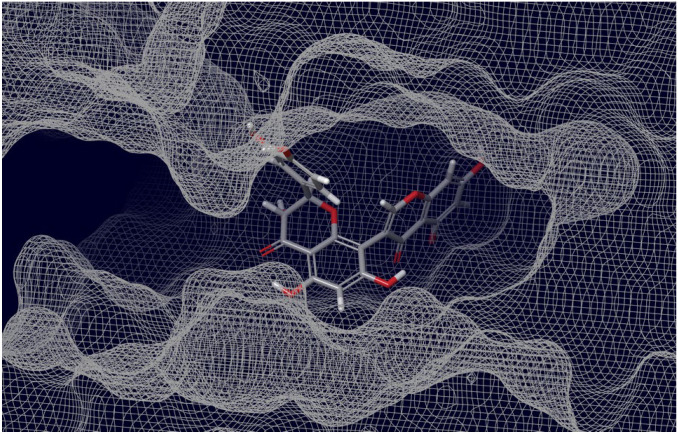
Interaction of ACE2 with Preussianone, the latter aligned with the *c* active site environment of the receptor complex (PDB ID: 7T9L).

As can be judged from the data presented in Table 4, in the series of the studied top-ligands, the docking energies are in the range of −9.98 to −6.21 kcal/mol. At that, one of the positions of the CNP0277806 ligand in the ACE2 binding pocket *c* turned out to be the most stable, with an IFD of −1677.2 kcal/mol. Analysis of the interactions of CNP0277806 with ACE2 residues showed that it is located deeply within the binding pocket of the ACE2, being in the cleft between the two *quasi*-subunits constituted by the His374-Tyr515 dyad, see Figure 10. This finding indicates the fact that CNP0277806 have the potential to covalently bind to amino acid residues at this region of 7T9L. This ability to interact with an angiotensin-converting enzyme 2 provides additional benefits in suppressing viral activity. It should be noted that, among the studied series of potential SARS-CoV-2 spike RBD–ACE2 inhibitors of all key ligands, CNP0277806 showed the best coordination with the 7T9L surface cavity with the strongest binding energy and, accordingly, values of the inhibition constant and IFD score. As a matter of fact, the best IFD score does not always correspond to the strongest binding energy, since the resulting IFD score is significantly affected by the Prime energy value, which, unlike the Glide Docking Score, can reach several tens of thousands of kilocalories [87,88]. Indeed,

IFD score = 1.0 × GlideScore + 0.05 × PrimeEnergy(1)

The molecule of CNP0277806 (Preussianone [89]) has a distributed network of seven HBs in the binding pocket *c* of the cleft of ACE2, including that between the oxygen atom of Gln522 and the hydrogen atom of the hydroxy group of the chromenone moiety; see Figure 8c and Figure 9c. The second and third HBs are formed between the hydrogen atom of the amino group of Arg518, carbonyl oxygen of Glu402, and one of the hydroxy groups of the central chromanone core of CNP0277806. The fourth HB is formed between the hydrogen atom of the amino group of Arg273 and the other hydroxy group of the chromanone moiety, while the fifth and sixth HBs are located between one hydroxy group of the pyrocatechol fragment and Pro346-Gln375. The seventh HB is found between the other hydroxy group of the same pyrocatechol moiety and the nitrogen atom of the aromatic ring of His345. Likewise, the residues Arg273 and Arg518 have *π*–alkyl contacts with the aromatic moiety of the chromanone fragment. On the other hand, there is also an interaction of the *π*-stacking sandwich-type between aromatic systems of His374 and the pyrocatechol fragment of CNP0277806.

The molecule CNP0332318 is located in the central contact zone *a* between the receptor-binding domain of spike and ACE2; see Figure 8a and Figure 9a. At the same time, it is stabilized by seven HBs, three of which are formed between the carbonyl oxygen atom of the acetophenone moiety and one of the aromatic protons of Tyr501(A), together with the hydrogen atoms of the amino groups Lys353(D) and Ser496(A). The fourth and fifth HBs are located between the protons of the aniline fragment and the carboxyl group of Glu37(D). The sixth HB is localized between the oxygen of the urea moiety and hydroxy group of Tyr453(A), while the seventh HB lies between the hydrogen of the amino group of Asn33(D) and one of the nitrogen atoms of the diazine fragment of CNP0332318. The additional stabilization of this ligand is possible due to the two *π*–alkyl contacts of the acetophenone fragment with the protons of the amino groups Arg403(A) and Lys353(D), as well as the *π*-stacking T-shaped-type interaction of the same fragment with the aromatic system of His34(D).

As for the ligand CNP0401960 of the binding domain *b*, five HBs can be noted here, two of which are located between the hydroxy group of the chromenone–pyranone core and Gln42(D); see Figure 8b and Figure 9b. The remaining three hydrogen bonds are formed between the carboxyl group of CNP0401960 and Lys444(A), Tyr449(A), and Gln42(D). As well as in the binding pocket *a*, the nonvalent *π*–alkyl and *π*-stacking parallel-displaced-type interactions of the protons of the amino group of Arg498(A) and the phenolic ring of Tyr449(A), respectively, are realized with the chromenone–pyranone aromatic system of the ligand under study. 

An extensive network of nine HBs has been realized in the binding site *d* between the receptor and the CNP0380471 ligand [90], as shown in Figure 8d and Figure 9d. The three of them are formed between Asn331 and Ile332 and one of the hydroxy groups of the chromenone moiety. The second hydroxy group of the same fragment forms two more HBs with Lys528 and Ser530, while the sixth and the seventh HBs are located between Lys529, Asp364, and the carbonyl oxygen atom and one of the aromatic hydrogens of the chromenone fragment, accordingly. The eighth HB is located between the hydroxy group of the dioxin core and the carboxyl oxygen atom of Cys336. Finally, the ninth HB arises between the hydroxy group of the methoxyphenol fragment and Asn343. In the course of binding of this ligand, the *π*–alkyl interaction arises between the aromatic system of the benzodioxine fragment of CNP0380471 and Lys529.

At the same time ligand CNP0340958 [91], which is Apocholic Acid, forms a more moderate set of interactions with RBD at the *e* binding site. As is seen in Figure 8e and Figure 9e, it is represented by six HBs, three of which are located between the hydroxy group of apocholic acid and Arg346 and Lys444. Two other HBs are formed between Asn354, Ser399, and the hydroxy group of the naphthalenole fragment. The final HB is located between the hydroxy group of the indenol fragment of the Apocholic Acid and Thr345.

Ligand CNP0393256 is quite well-known [92] and is none other than Hesperetin; it is stabilized at binding site *f* on the surface of ACE2 primarily by a network of the non-valent *π*-interactions, as shown in Figure 8f and Figure 9f. In particular, two strong *t*-stacking contacts are realized between the aromatic system of the methoxyphenol moiety of Hesperetin and both aromatic cycles of the indole moiety of Trp610. Two more *π*–alkyl interactions are formed between Arg482 and two cycles of Hesperetin. There is also a similar contact with Lys475. Hydrogen bonds are represented by the contacts of Thr608 with the methoxy group and those of His493 with the hydroxy groups of Hesperetin.

The CNP0125042 ligand, which is one of the derivatives of the Xanthines class, forms a strong network of non-valent interactions in the *g* binding pocket in the small hydrophobic cavity near the cleft of ACE2. The hydrogen bonding is shown in more detail in Figure 8g and Figure 9g. Out of the seven HBs, three bonds are located between Asn103 and His195 and both oxygen atoms of the pyrimidinedione cycle. The fourth HB is located between the NH proton of the same cycle and Asn194. The fifth HB is located between the proton of the hydroxy group of Tyr196 and the nitrogen atom of one of the diazole rings of CNP0125042. The sixth and seventh HBs are formed between the protons of the amide group of CNP0125042 and Gly205, Glu208. At that, *π*–alkyl interactions are represented by three contacts of the terminal amino group of Arg219 with three aromatic rings of the studied ligand, while *t*-stacking displaced-type is realized between the azole ring and Tyr196.

It is interesting to note that, in all cases considered herein (as has already been indicated by several authors), the base for the stabilization of potential inhibitors in the main binding interface of RBD–ACE2 (*a*) is a distributed network of hydrogen bonds, predominantly those involving residues Tyr453(A), Ser496(A), Asn33(D), and Lys353(D). On the other hand, it is a non-valence coordination of the aromatic systems of ligands with Arg403(A), Lys353(D), and His34(D) within the formation of stable *π*-contacts.

The results of the present study demonstrated that natural products from the top-ligands set are predicted to effectively fit into the main considered active sites of RBD, ACE2, and RBD–ACE2 with high affinity, which was confirmed by the IFD protocol.

## 3. Materials and Methods

### 3.1. Preparation of Protein for Docking and Grid Generation

The cryo-EM structure of the SARS-CoV-2 Omicron spike protein in complex with human ACE2, (focused refinement of RBD and ACE2) with resolution 2.66 Å, was obtained from the Protein Data Bank (PDB ID: 7T9L [38]). The 7T9L macromolecule contains two chains, A (RBD-S1 of the spike glycoprotein) and D (processed ACE2), that are binding through a specific interface. This complex of chains was used as a receptor for protein preparation with using Schrödinger Maestro 11.5 [93]. The co-factors (2-acetamido-2-deoxy-*β*-D-glucopyranose) and water molecules were removed, and absent hydrogen atoms were added. Further, the receptor structure was refined using the PROPKA protocol [94] at pH = 7.0 and then energy-minimized within the OPLS3 force field. The receptor grids were generated using the appropriate sets of residues as the centroids for each of the studied binding sites *a*–*g*, while the size of each of the grid boxes was 30 × 30 × 30 Å.

### 3.2. Preparation of Ligands

The 2D structures of ligands downloaded in SDF format from the COCONUT natural compound database [37] were initially filtered according to Lipinski’s rule [95]. Further, ligands containing reactive functional groups were removed [96]. At the next stage, for the filtered ligands of sets *a*–*g*, low-energy tautomeric states for the target pH = 7 ± 2 were generated using the Epik module [97].

### 3.3. Development of Pharmacophore Models

Pharmacophore hypotheses were generated based on the analysis of residues of the binding domains *a*–*g* of the receptor (ACE2, RBD and RBD-ACE2; PDB ID: 7T9L). For each binding site, 4 pharmacophore models were developed: a model based on ligand–protein interactions, a model using the receptor cavity, a model taking into account multiple ligands, and finally, a model combining the previous three—the so-called merge model. Hypotheses were generated using the Phase module [98]. In each case, from 4 to 7 features of the pharmacophore were used, including:

- Hydrogen bond acceptor (A);
- Hydrogen bond donor (D);
- Aromatic ring (R);
- Positive ionizable (P);
- Negative ionizable (N);
- Hydrophobic center (H).

Since the characteristics of the hydrogen bond donor and acceptor are of a vector nature, they determine the direction of electron exchange.

### 3.4. Molecular Docking Simulations

Molecular docking using the Schrödinger Maestro 11.5 [93] was employed to rapidly determine the ligand-binding poses and affinity to human ACE2 and SARS-CoV-2 spike RBD/RBD–ACE2. Molecular docking at the stage of the preparation of identical structures for the formation of a library of NP was carried out at the extra-precision level using the glide module [99]. For each one of the tautomers formed at the stage of ligand preparation, 50 conformational states were generated with an energy window for ring sampling of 2.5 kcal/mol. In this case, the ligand sampling method was chosen as flexible, and the maximum minimization steps were 100. Post-docking minimization included 10 poses per ligand, with a threshold for rejecting minimized pose of 0.5 kcal/mol.

Final Induced Fit Docking was performed for key ligands with the best Phase Screen Scores of the pharmacophore hypotheses 1–4 for all binding sites *a*–*g*. The docking protocol was assigned as the standard for flexible protein and ligand, generating up to 20 poses within the OPLS3 force field. The refinement of residues was carried out in the framework of the Prime module [100] within 5.0 Å of ligand poses. The redocking procedure was performed with the studied ligands in their respective cavities within 30 kcal/mol of their lowest energy structure. At the final stage, the best docking modes of all compounds were selected from their conformations based on the docking score, as well as on significant non-valence interactions observed with the receptor. The interaction analyses were performed using Schrödinger Maestro 11.5.

## 4. Conclusions

In the present study, we proposed and used a comprehensive approach based on the generation of pharmacophore models and subsequent Induced Fit Docking to identify potential inhibitors of the main binding sites of the Omicron SARS-CoV-2 RBD(S1)–ACE2 complex (PDB ID: 7T9L) among a number of natural products of different origins.

The pharmacophore models were created on the basis of four types of hypotheses, namely: receptor–ligand, receptor cavity, multiple ligands, and the merged hypothesis. Each model of each of the binding domains was used to conduct a virtual screening of the generated libraries from about 25,000 natural compounds from the COCONUT database. The found key ligands were then used for extra-precision molecular docking in the framework of the IFD protocol. The results of the performed molecular docking of the established structures of the top ligands were used to study the binding interactions in the main active centers of the RBD–ACE2 complex. For several natural compounds with high affinity for the receptor of interest, non-polar, *π*-stacking, and other electrostatic interactions were found to stabilize these ligands in the binding pocket. Undoubtedly, one of the main roles was played by the distributed networks of the ligand–receptor hydrogen bonds.

As a result of this study, it was found that Preussianone, which is a natural extract from the leaves of *Garcinia preussii*, showed the best affinity for the binding pocket *c* in the cleft of ACE2. Several other natural products being tested in this study, such as CNP0332318, CNP0401960, CNP0380471, Apocholic Acid, Hesperetin, and CNP0125042, also showed better affinity for their binding domains compared to the rest of the tested ligands.

The selected potential inhibitor candidates identified in this work showed improved interaction energies relative to the RBD–ACE2 complex, providing increased specificity due to the additional hydrogen bonding with the active site residues. It is expected that the presented results will stimulate further research aimed at the development of specialized drugs against the SARS-CoV-2 virus.

## Figures and Tables

**Table 1:** Potential inhibitors of the basic domains *a*–*g* of SARS-CoV-2 Spike RBD–ACE2 previously identified by in silico investigations.

Drug Name	Source	Pharmacological Function(s)	Binding Energy Score, kcal/mol *^a^*	References
binding domain: *a*				
7-Methyl-guanosine-5′-triphosphate-5′-guanosine	synthetic	a biomarker of some types of cancer	−9.1	[40]
8-Bromo-adenosine-5′-monophosphate	synthetic	inhibition of inosine monophosphate dehydrogenase in *Escherichia coli*	−8.1	[40]
Acalabrutinib	synthetic	inhibition of mantle cell lymphoma and chronic lymphocytic leukemia	−7.2	[66]
Acitretin	synthetic	treatment of severe psoriasis and other skin disorders in adults	−9.6	[67]
Adenosine-2′-5′-diphosphate		agonist activity at P2Y1 receptor in turkey erythrocyte membranes	−8.6	[40]
Alpinumisoflavone	*Erythrina lysistemon*	antischistosomal activity	−10.7	[56]
Cladribine	synthetic	a medication used to treat hairy cell leukemia and B-cell chronic lymphocytic leukemia	−7.9	[40]
Clofarabine	synthetic	treating relapsed or refractory acute lymphoblastic leukaemia	−7.2	[40]
Curcumin	genus *Curcuma*	antiinflammatory, antitumor activity	−9.0	[48]
Demethylzeylasteral	*Tripterygium wilfordii Hook F.*	androgen receptor in human LNCAP cells	ND	[44]
Dexamethasone	synthetic	anti-inflammatory and immunosuppressant effects; treating arthritis, severe allergies, asthma, and certain types of cancer	−6.5	[68]
Dieckol	*Eisenia bicyclis*	antithrombotic and profibrinolytic activities	−8.1	[69]
Dimethylcurcumin	synthetic	antiandrogen activity	−11.2	[48,70]
Dithymoquinone	*Nigella sativa*	therapeutic of inflamation	−8.6	[51]
Epigallocatechin-3-gallate	*Camellia sinensis*	antioxidant effects, cancer chemoprevention, improving cardiovascular health, enhancing weight loss	ND	[49,71,72]
Ergocalciferol (Vitamin D2)	Fish oil	a dietary supplement to prevent and treat vitamin D deficiency	−14.8	[73]
Evans Blue	synthetic	a negative allosteric modulator of the AMPA and kainate receptors and an inhibitor of vesicular glutamate transporters	ND	[49]
Fludarabine	synthetic	a chemotherapy medication used in the treatment of leukemia and lymphoma	−7.0	[40]
Glycyrrhizin	*Glycyrrhiza radix*	emulsifier and gel-forming agent in foodstuffs and cosmetics	−9.0	[20]
Hesperidin	*Citrus aurantium*	inhibitor of the TRPM3 channels	−9.5	[48]
Indacaterol	synthetic	an ultra-long-acting beta-adrenoceptor agonist used for the treatment of chronic obstructive pulmonary disease in patients with asthma	−8.1	[53]
Kobophenol A	*Caragana chamlagu*	inhibitor of acetylcholinesterase	−11.1	[45]
Levodopa	*Mucuna pruriens*	an amino acid precursor of dopamine with antiparkinsonian properties	−6.1	[67]
Luteolin	*Reseda luteola*	a principal yellow dye compound	−7.8	[53]
Parvisoflavone B	*Erythrina schliebenii*	antitubercular and cytotoxic activity	−10.7	[56]
Rutin	*Fagopyrum esculantum*	antioxidant and cytoprotective properties	−7.9	[21]
Taraxerol	*Taraxacum officinale*	antiinflammatory activity	−7.5	[23]
Tazarotene	synthetic	treatment of plaque psoriasis and acne and a therapeutic for photoaged and photodamaged skin	−6.1	[67]
Tretinoin	a natural derivative of vitamin A	treatment of acne and follicular keratosis and the curing of acute promyelocytic leukemia	−6.0	[67]
Ursodeoxycholic acid	genus *Ursus*	treatment of several diseases of the liver or bile ducts	−7.0	[53]
Velpatasvir	synthetic	the NS5A inhibitor used in the treatment of hepatitis C infection	−11.1	[74]
Venetoclax	synthetic	a medication used to treat adults with chronic lymphocytic leukemia, small lymphocytic lymphoma, and acute myeloid leukemia	ND	[49,75,76,77]
Vitamin B12	*Propionibacterium shermanii*	hematopoiesis, neural metabolism, DNA and RNA production	−7.6	[22]
Vitamin K2	*Mycobacterium tuberculosis*	a common form of vitamin K, primarily necessary for the body to carry out vital processes, cleaning blood vessels, and blood clotting	−9.5	[78]
binding domain: *b*				
Acetoside	*Olea europaea*	antioxidant, anti-inflammatory activity	−8.5	[21]
Amentoflavone	*Ginkgo biloba*	inhibitor of CYP3A4 and CYP2C9, which are enzymes responsible for the metabolism of some drugs in the body; it is also an inhibitor of human cathepsin B	−8.5	[26]
Arbidol	synthetic	a broadspectrum respiratory antiviral drug	−7.7	[58]
Celastrol	*Tripterygium wilfordii*	antitumor action, inhibitor of inflammatory and human prostate cancer activities	−8.3	[26]
Dioscin	*Ophiopogon intermedius*	antitumor, antimicrobial, anti-infammatory, antioxidative, and tissue-protective activities	−8.9	[26]
Epimedin C	*Herba epimedii*	treatment of cardiovascular disease and bone loss	−8.1	[26]
Epitheaflavin monogallate	*Camellia sinensis*	antitoxicant, antioxidant, and antiinflammatory activity	−7.5	[21]
Saikosaponin	*Bupleurum chinense*	treatment of hepatitis in Chinese herbal medicine	−9.1	[26]
Solanine	*Solanum nigrum*	fungicide, antimicrobial and pesticide properties	−9.5	[21]
binding domain: *c*				
Anabsinthin	*Artemisia absinthium* L.	inhibition of the human immunodeficiency virus 1 (HIV1) protease, treating acute bacillary dysentery	−12.5	[25]
Atazanavir	synthetic	the inhibitor of the HIV protease; selectively inhibits the virus-specific processing of viral Gag-Pol proteins in the HIV-infected cells, preventing the infection of other cells	−12.4	[62]
Baicalin	*Scutellaria baicalensis*	antioxidant, anti-inflammatory, and anti-apoptosis properties	−8.5	[20]
*β*-Sitosterol	*Solanum trilobatum*	reduction of benign prostatic hyperplasia and blood cholesterol levels	−10.9	[62]
Caflanone	*Cannabis sativa*	selective activity against the human coronavirus (COVID-19) disease; vasorelaxant activity against phenylephrine-induced contraction in rat aorta	−7.9	[61]
Chloroquine	genus *Cinchona*	a medication used to prevent and treat malaria	−6.5	[52]
Demethylzeylasteral	*Tripterygium wilfordii*	antitumor effects in a variety of cancers, inhibits the proliferation, migration, and invasion of gastric cancer cells	ND	[44]
Epitheaflavin monogallate	*Camellia sinensis*	cancer-fighting chemical when combined with cisplatin against ovarian cancer cells	−7.5	[21]
Ertapenem	synthetic	a carbapenem antibiotic medication used for the treatment of infections of the abdomen, the lungs, the upper part of the female reproductive system, and the diabetic foot	−8.8	[41]
Flavin adenine dinucleotide	cow milk	a cofactor for cytochrome-*b5* reductase, the enzyme that maintains hemoglobin in its functional reduced state	−8.6	[41]
Indacaterol	synthetic	an ultra-long-acting beta-adrenoceptor agonist licensed for the treatment of chronic obstructive pulmonary disease	−8.1	[41,53]
Kaempferol	*Lycopodiella inundata*	a multipotential neuroprotective action through the modulation of several proinflammatory signaling pathways	−10.4	[62]
Ledipasvir	synthetic	a direct acting antiviral medication used as part of combination therapy to treat chronic hepatitis C and exhibiting many pharmacological activities	−9.1	[41]
Naringenin	genus *Citrus*	inhibition of some drug-metabolizing cytochrome P450 enzymes including CYP3A4 and CYP1A2	−6.4	[79]
Nicotianamine	*Glycine max*	potent inhibitor of the angiotensin-converting enzyme ACE2	−5.1	[20]
Raltegravir	synthetic	a potent CYP3A inhibitor decreasing the amount of human immunodeficiency virus in human blood	−9.1	[41]
Stigmasterol	*Ophiopogon japonicus*	maintaining the structure and physiology of cell membranes	−9.8	[62]
binding domain: *d*				
Chrysin	*Scutellaria baicalensis*	antivirus and antiinflammatory properties	−6.5	[53]
Glycyrrhizin	*Glycyrrhiza radix*	antihepatotoxic activity	−9.0	[20]
Linoleic acid	*Carthamus tinctorius*	one of two essential fatty acids for humans, who must obtain it through their diet	−6.8	[80]
Myricetin 3-(4″-galloylrhamnoside)	*Limonium* species	an excellent source of phytosterols and flavonoids	−8.3	[24]
Myricetin 3-rhamnoside	*Newtonia buchananii*	active against *B. cereus*, *E. coli*, and *S. aureus*	−8.5	[24]
Pelargonidin	genus *Geranium*	a type of plant pigment producing a characteristic orange color, which is used in food and industrial dyes	−7.7	[59]
binding domain: *e*				
Betulinic acid	*Betula pubescens*	a naturally occurring pentacyclic triterpenoid providing antiretroviral, antimalarial, and anti-inflammatory properties, as well as a more recently discovered potential as an anticancer agent	−8.1	[56]
Canrenone	active metabolite of spironolactone	an antimineralocorticoid and active metabolite of spironolactone used in the treatment of primary hyperaldosteronism	−7.9	[56]
Glycyrrhizin	*Glycyrrhiza radix*	a component of licorice, causes apparent mineralocorticoid excess through the inhibition of the enzyme 11-β-hydroxysteroid dehydrogenase	−9.0	[20]
Oleanolic acid	*Olea europaea*, *Rosa woodsii*	exhibiting antitumor and antiviral properties together with weak anti-HIV and weak anti-HCV activities in vitro	−8.2	[25,56]
Potassium canrenoate	synthetic	an aldosterone antagonist of the spirolactone group, metabolizing to active canrenone	−6.9	[56]
binding domain: *f*				
Hesperetin	*Citrus aurantium*	inhibitor of the M^pro^ of SARS-coronaviruses	−9.1	[20]
Scutellarin	*Erigeron breviscapus*	antiplatelet and anticoagulation properties	−14.9	[20]
binding domain: *g*				
2-vinyl-4*H*-1,3-dithiine	*Allium sativum*	affecting the vascular smooth muscle cells isolated from spontaneous hypertensive rats	−14.0	[64]
Abemaciclib	synthetic	a medication for the treatment of advanced or metastatic breast cancers	−9.9	[41,67]
Allyl disulfid	*Allium sativum*	providing antioxidative, antiviral, neuroprotective, antiparasitic, anticancer, and antihyperlipidemic activities	−15.3	[64]
Allyl methyl trisulfide	*Allium chinense*, *Mansoa alliacea*	used as flavoring agent and tumor inhibitor	−14.4	[64]
Allyl propyl trisulfide	*Azadirachta indica*	used in food additives and flavors	−14.0	[64]
Caffeic acid phenethyl ester	Propolis	antimitogenic, anticarcinogenic, anti-inflammatory, and immunomodulatory properties in vitro	−6.5	[81,82]
Chrysin	*Passiflora caerulea*	an ingredient in dietary supplements	−7.1	[53]
Cianidanol	*Salix atrocinerea*, *Visnea mocanera*	an antioxidant flavonoid, occurring especially in woody plants	−9.5	[83]
Diallyl tetrasulfid	synthetic	shown to selectively kill cancerous cells in the prostate and breast, leaving healthy cells unharmed; providing also antioxidant, anti-inflammatory, and anti-apoptotic effects; and a promising treatment for cardiac arrhythmias	−14.5	[64]
Flavin adenine dinucleotide	cow milk	a redox-active coenzyme associated with various proteins, which is involved with several enzymatic reactions in metabolism	−9.9	[41]
Pinocembrin	*Turnera diffusa*	antioxidant, a drug to treat cerebral ischemia, intracerebral hemorrhage, neurodegenerative diseases, cardiovascular diseases, and atherosclerosis	−7.8	[63]
Ponatinib	synthetic	treatment of chronic myeloid leukemia and chromosome-positive acute lymphoblastic leukemia, a multi-targeted tyrosine-kinase inhibitor	−9.9	[41]
Saquinavir	synthetic	an antiretroviral drug used to treat or prevent HIV/AIDS	−11.7	[41,62,67]
Siponimod	synthetic	a selective sphingosine-1-phosphate receptor modulator for oral use for multiple sclerosis	−9.9	[41,67]
Ursodeoxycholic acid	genus *Ursus*	used as therapy in primary biliary cholangitis; for intrahepatic cholestasis of pregnancy; has been suggested to be an adequate treatment of bile reflux gastritis	−8.7	[45,53,65,84]

*^a^* The strongest binding energy; ND—no data available.

**Table 2:** A brief description of the binding domains *a*–*g*.

Binding Domain	Peculiarities of Domain	Residues
*a*	Central contact area RBD with ACE2	**RBD**: Glu406, Arg403, Ser496, His505 **ACE2**: His34, Asp30, Lys353, Thr27
*b*	Hydrophobic pocket beside the interaction interface of RBD-ACE2	**RBD**: Tyr449, Leu452, Ala352
*c*	Catalytic cleft of ACE2	**ACE2**: Thr371, Glu406, Arg273, His345, Asn149
*d*	Bent FA hydrophobic tube of RBD	**RBD**: Leu368, Leu387, Phe388, Phe342, Ile434, Phe377, Phe338, Tyr365, Ala372
*e*	*β*-sheet in the core of the RBD	**RBD**: Lys440, Ser438, Arg346, Asp442, Val445, Tyr451
*f*	Deepening pocket at the ACE2 surface	**ACE2**: Arg482, Glu495
*g*	Hydrophobic pocket alongside the cleft of ACE2	**ACE2**: Ser511, Tyr196, Gln102, Glu208, Pro565, Trp 566, Ala 396, Gln 98, Leu91

**Table 3:** Screening parameters for the key and top-ligands according to the pharmacophore models *a*–*g*.

Library Size, Cmpds.	Hypothesis	Structure of Hypothesis	Key Ligand	Matched Ligand Sites	Phase Screen Score	Top Ligand (IFD)
binding domain: *a*						
2438	a1	ADDDDNR	CNP0260198	DDNR	1.418	CNP0332318
			CNP0141274	ADDR	1.292	
	a2	AADDDNR	CNP0363429	ADDR	1.606	
			CNP0123143	ADDR	1.498	
			CNP0332318	AADD	1.477	
	a3	AAADR	CNP0224071	AAADR	2.743	
		AAARR	CNP0274243	AAARR	2.704	
		AAARR	CNP0322514	AAARR	2.701	
	a4	AAADDDNNRR	CNP0305586	AADR	1.320	
			CNP0429890	ADRR	1.291	
binding domain: *b*						
3632	b1	ADDRRRR	CNP0129813	ADRR	1.811	CNP0401960
	b2	AAHNNNR	CNP0129813	AHN	1.516	
			CNP0401960	AHNR	1.412	
	b3	ADHR	CNP0131499	ADHR	1.898	
		AADHR	CNP0146455	AADHR	1.860	
		AADHR	CNP0403928	AADHR	1.808	
	b4	ADDNRRRR	CNP0128506	ADNR	1.393	
binding domain: *c*						
3657	c1	ADDDRRR	CNP0277806	DDDRR	1.665	CNP0277806
			CNP0302437	DDDRR	1.646	
			CNP0318431	DDRR	1.622	
			CNP0129813	ADRR	1.620	
	c2	AAADDRR	CNP0409641	AARR	1.604	
			CNP0271209	AAADRR	1.555	
			CNP0406372	AADR	1.547	
			CNP0131497	AAAR	1.512	
	c3	AAARR	CNP0437810	AAARR	2.781	
	c4	AAADDDRRR	CNP0168889	AADDRR	1.409	
			CNP0153057	AADDRR	1.394	
			CNP0310325	ADDDRR	1.393	
binding domain: *d*						
4953	d1	DDRRR	CNP0182350	DDRR	1.608	CNP0380471
			CNP0191402	DDRR	1.508	
	d2	AADDHNR	CNP0429546	AAHNR	1.453	
	d3	ADDRRR	CNP0318928	ADDRRR	2.630	
			CNP0140035	ADDRRR	2.586	
			CNP0380471	ADDRRR	2.517	
	d4	AADDDHNRRR	CNP0204419	AAHNR	1.327	
			CNP0348217	AADR	1.220	
binding domain: *e*						
4847	e1	HHN	CNP0161706	HHN	1.594	CNP0340958
	e2	AADNRRR	CNP0340958	AADN	1.348	
	e3	AHHHN	CNP0287935	AHHHN	2.688	
	e4	AADHNRRR	CNP0360609	AAHH	1.533	
			CNP0364398	AAHH	1.504	
			CNP0329427	AAHH	1.475	
binding domain: *f*						
4431	f1	ADRR	CNP0393256	ADRR	2.692	CNP0393256
	f2	ADDDRRR	CNP0104690	ADRR	1.540	
			CNP0148806	ADRR	1.505	
			CNP0122888	ADDRR	1.499	
	f3	AAADRR	CNP0393256	AAADRR	2.981	
	f4	AADDDRRRR	CNP0302437	ADRRR	1.481	
			CNP0347670	ADRR	1.472	
binding domain: *g*						
620	g1	ADDNRRR	CNP0342552	ADRR	1.413	CNP0125042
			CNP0202472	ADRR	1.175	
			CNP0176937	ADRR	1.170	
	g2	ADDDDDD	CNP0391500	ADDD	1.398	
			CNP0005103	ADDD	1.389	
			CNP0176937	ADDD	1.352	
	g3	AADRR	CNP0125042	AARR	2.126	
	g4	AADDDDNRR	CNP0071844	ADRR	1.298	

**Table 4:** Induced Fit Docking results for the SARS-CoV-2 RBD–ACE2 of top inhibitors.

Binding Domain	Ligand	Binding Energy, kcal/mol	IFD Score, kcal/mol	Type of Interactions of Residues
*a*	CNP0332318	−6.71	−1673.0	**H-bond:** Tyr453(A), Ser496(A), Tyr501(A), Asn33(D), Glu37(D), Lys353(D) ***π*-alkyl:** Arg403(A), Lys353(D) ***t*-stacking:** His34(D)
*b*	CNP0401960	−7.93	−1672.3	**H-bond:** Lys444(A), Tyr449(A), Gln42(D) ***π*-alkyl:** Arg498(A) ***π*-stacking:** Tyr449(A)
*c*	CNP0277806	−9.98	−1677.2	**H-bond:** Arg273(D), His345(D), Pro346(D), Gln375 (D), Glu402(D), Arg518(D), Gln522(D) ***π*-alkyl:** Arg273(D), Arg518(D) ***π*-stacking:** His374(D)
*d*	CNP0380471	−8.68	−1673.6	**H-bond:** Asn331(A), Ile332(A), Cys336(A), Asn343(A), Asp364(A), Lys528(A), Lys529(A), Ser530(A) ***π*-alkyl:** Lys529(A)
*e*	CNP0340958	−6.21	−1671.4	**H-bond:** Thr345(A), Arg346(A), Asn354(A), Ser399(A), Lys444(A)
*f*	CNP0393256	−6.56	−1671.8	**H-bond:** His493(D), Thr608(D) ***π*-alkyl:** Lys475(D), Arg482(D) ***t*-stacking:** Trp610(D)
*g*	CNP0125042	−9.16	−1676.5	**H-bond:** Gln98(D), Asn103(D), Gly104(D), Asn194(D), His195(D), Tyr196(D), Gly205(D), Glu208(D) ***π*-alkyl:** Arg219(D) ***t*-stacking:** Tyr196(D)

## Data Availability

All data are contained within the paper and Supplementary Materials.

## Supplementary Materials

The following supporting information can be downloaded at: https://www.mdpi.com/article/10.3390/molecules27248938/s1, Figures S1–S24: Pharmacophore models of *a*, *c*–*g*.
