# Supplementary material for: The Development of Pharmacophore Models for the Search of New Natural Inhibitors of SARS-CoV-2 Spike RBD–ACE2 Binding Interface
Source: Molecules. 2022 Dec 15;27(24):8938. doi: 10.3390/molecules27248938 (PMC9788546; doi:10.3390/molecules27248938)
Supplement: Supplementary file 1 [file molecules-27-08938-s001.zip › molecules-2063547-supplementary.pdf]

SUPPLEMENTARY MATERIALS

The development of pharmacophore models for the search of new natural inhibitors of SARS-CoV-2 spike RBD-ACE2 binding interface

Valentin A. Semenov* and Leonid B. Krivdin*

A. E. Favorsky Irkutsk Institute of Chemistry, Siberian Branch of the Russian Academy of Sciences, Favorsky St. 1, 664033 Irkutsk, Russia

Correspondence

Valentin A. Semenov, A. E. Favorsky Irkutsk Institute of Chemistry, Siberian Branch of the Russian Academy of Sciences, Favorsky St. 1, 664033 Irkutsk, Russia. e-mail: semenov@irioch.irk.ru

TABLE OF CONTENTS

Developed pharmacophore models for the main binding sites *a, c-g* of the SARS-CoV-2 spike RBD(S1)-ACE2 complex (PDB ID: 7T9L). 3

Binding pocket: *a* 3

Binding pocket: *c* 6

Binding pocket: *d* 9

Binding pocket: *e* 12

Binding pocket: *f* 15

Binding pocket: *g* 18

Developed pharmacophore models for the main binding sites *a*, *c-g* of the SARS-CoV-2 spike RBD(S1)-ACE2 complex (PDB ID: 7T9L).

Binding pocket: *a*

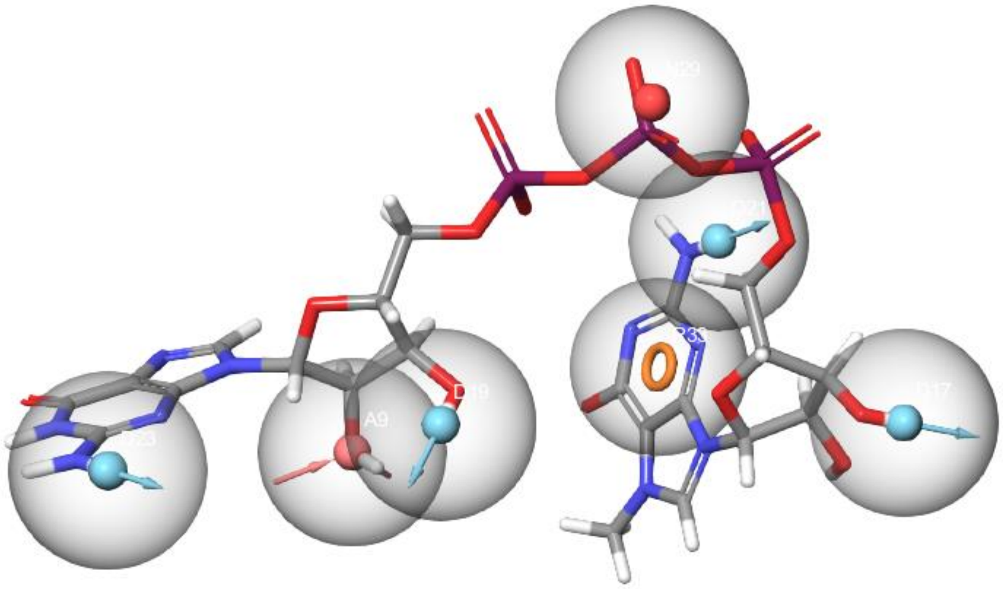

Figure S1. Pharmacophore model of the RBD site *a* of spike protein generated by the Receptor-ligand method.

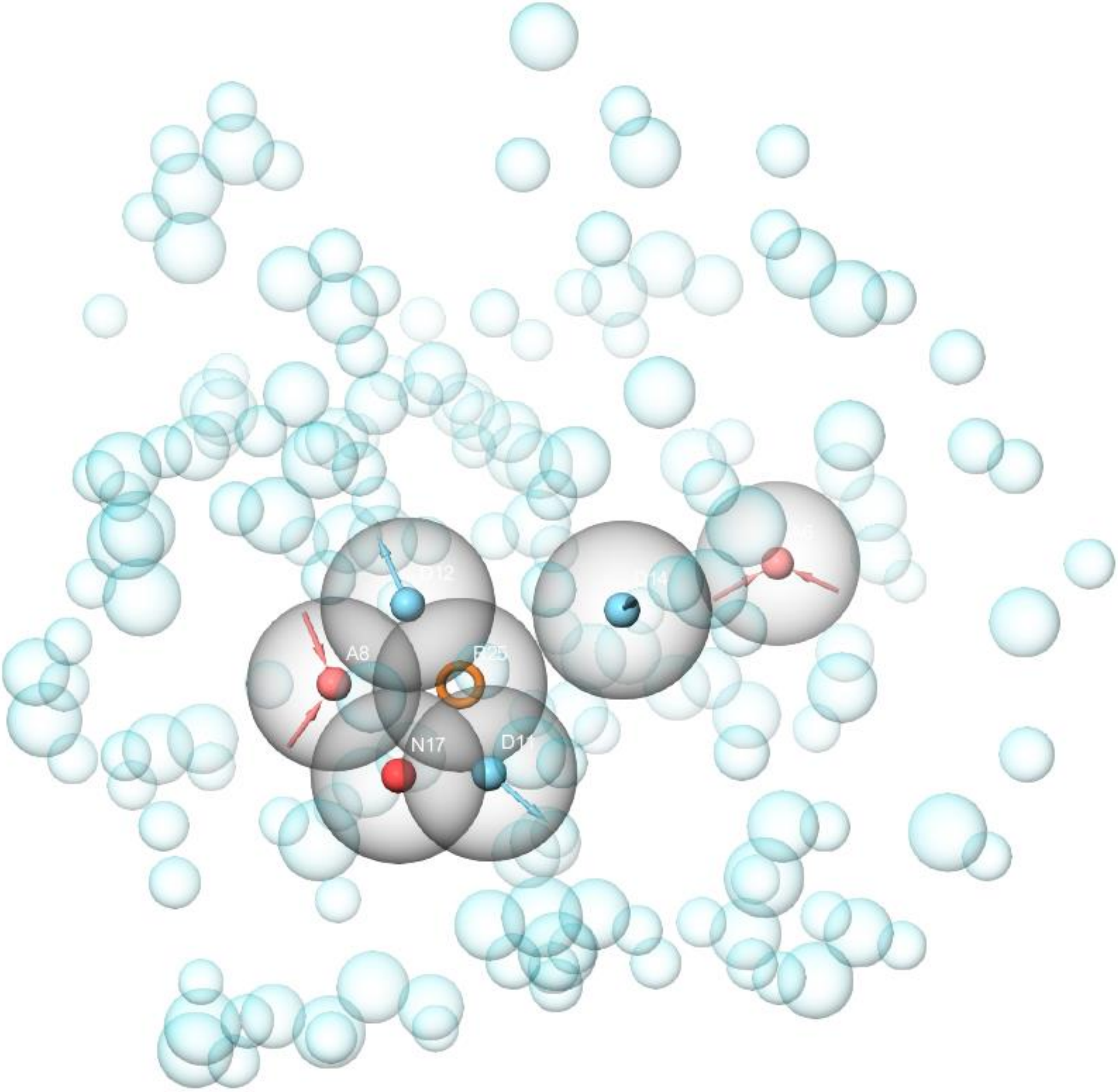

Figure S2. Pharmacophore model of the RBD site *a* of spike protein generated by the Receptor-cavity method.

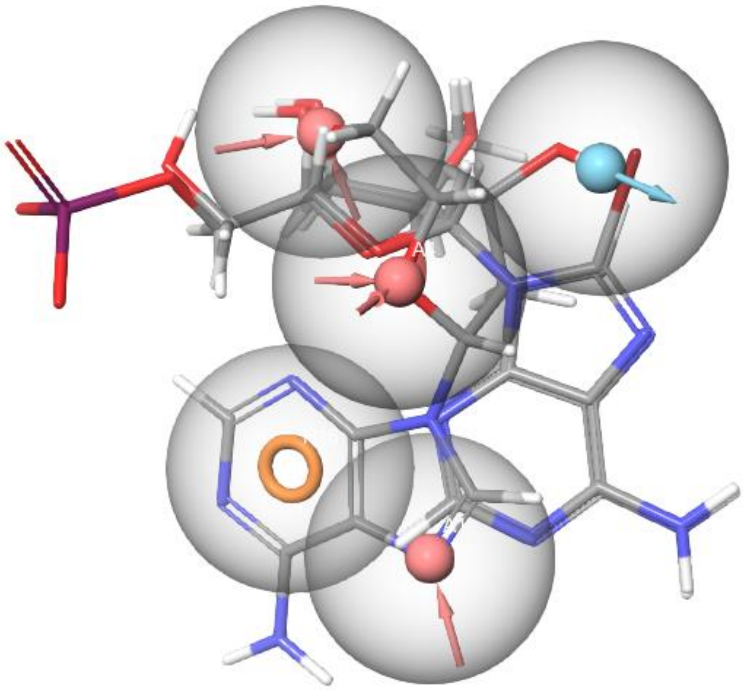

Figure S3. Pharmacophore model of the RBD site *a* of spike protein generated by the Multiple ligands method.

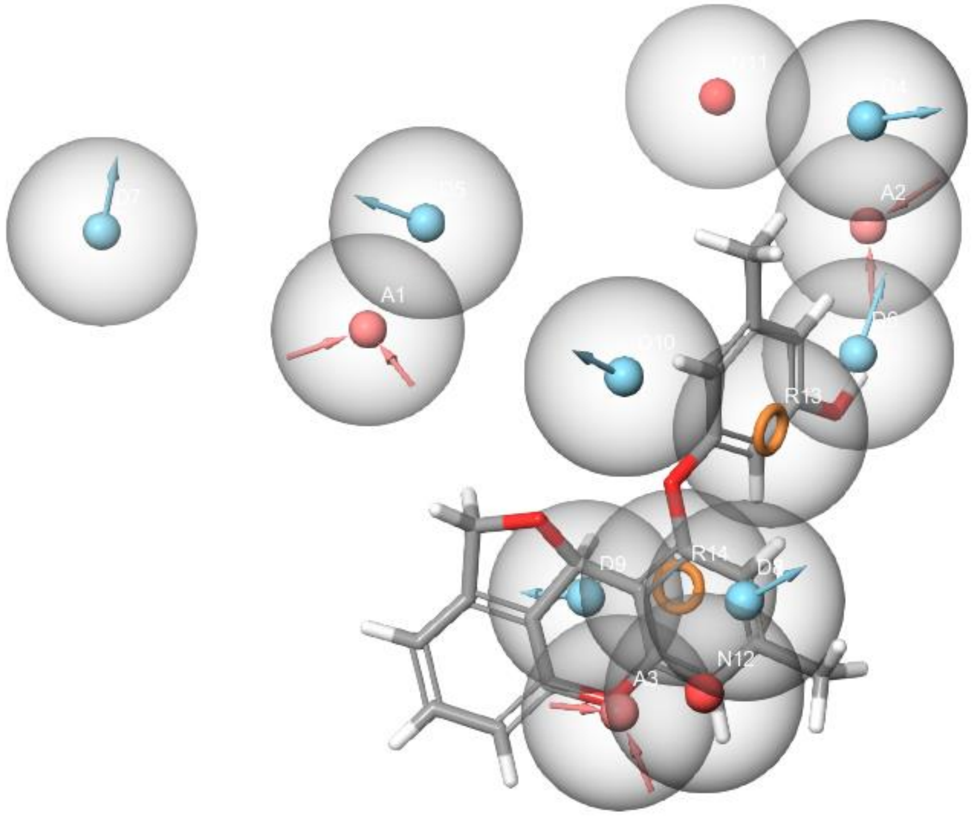

Figure S4. Pharmacophore model of the RBD site *a* of spike protein generated by the Merged hypothesis method.

Binding pocket: *c*

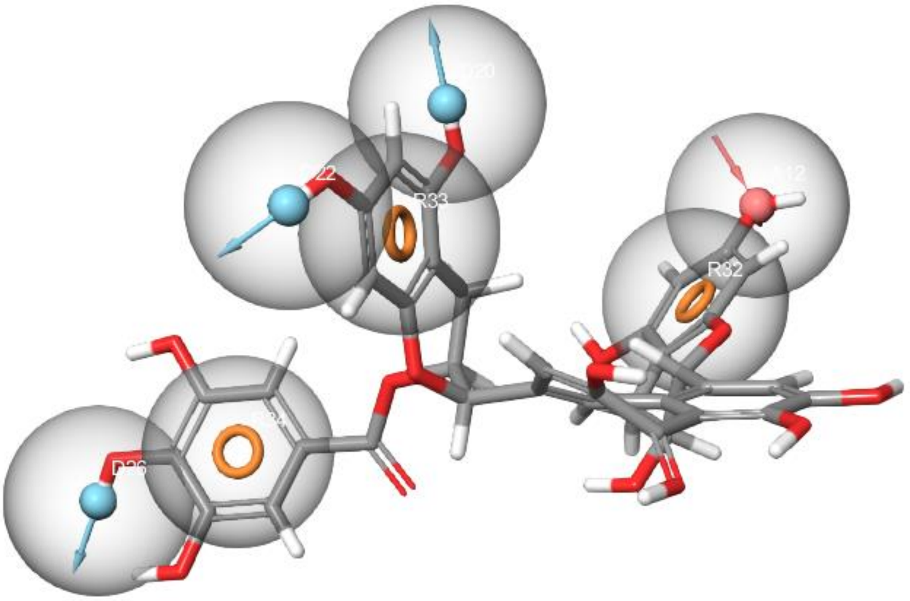

Figure S5. Pharmacophore model of the RBD site *c* of spike protein generated by the Receptor-ligand method.

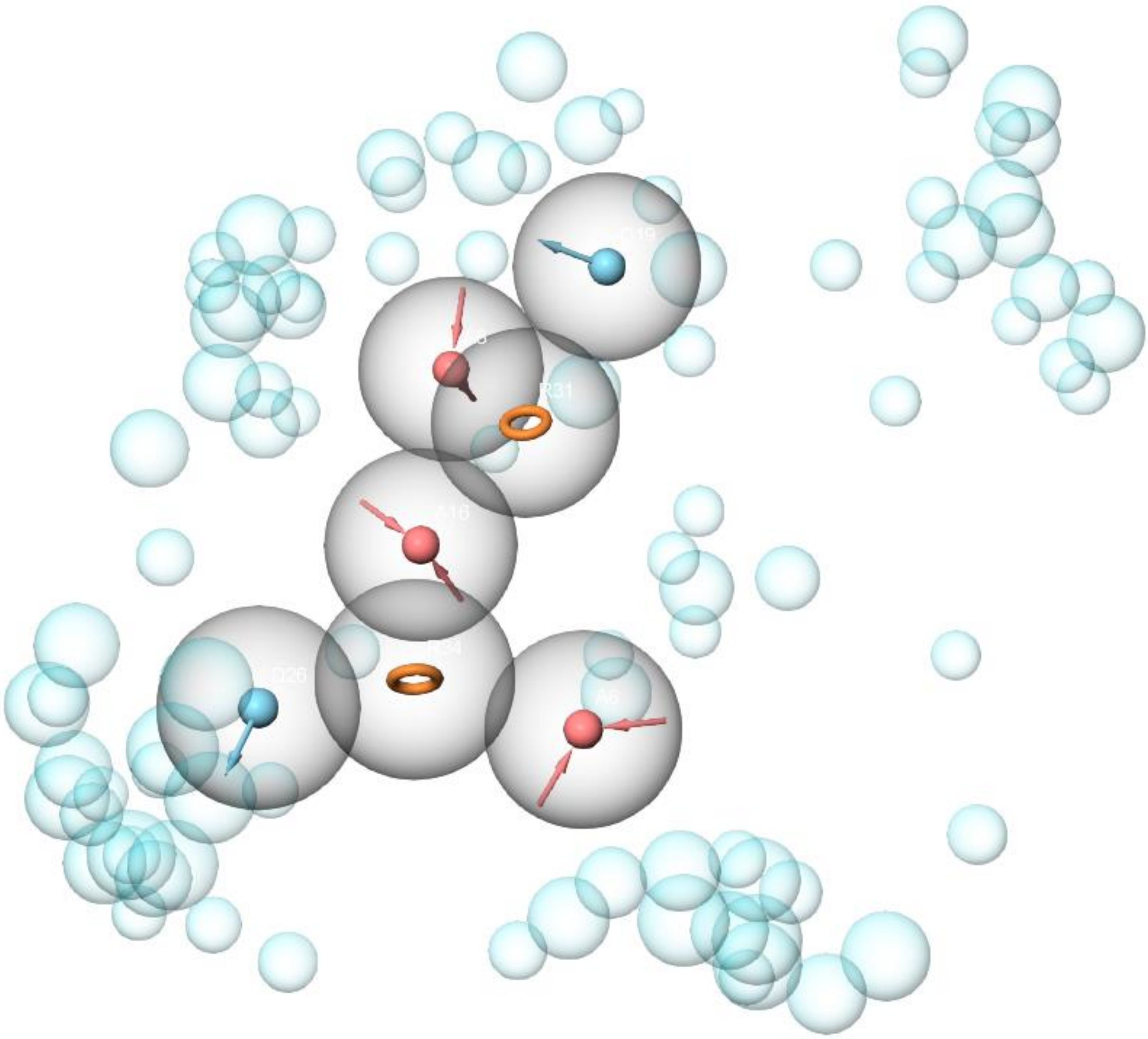

Figure S6. Pharmacophore model of the RBD site *c* of spike protein generated by the Receptor-cavity method.

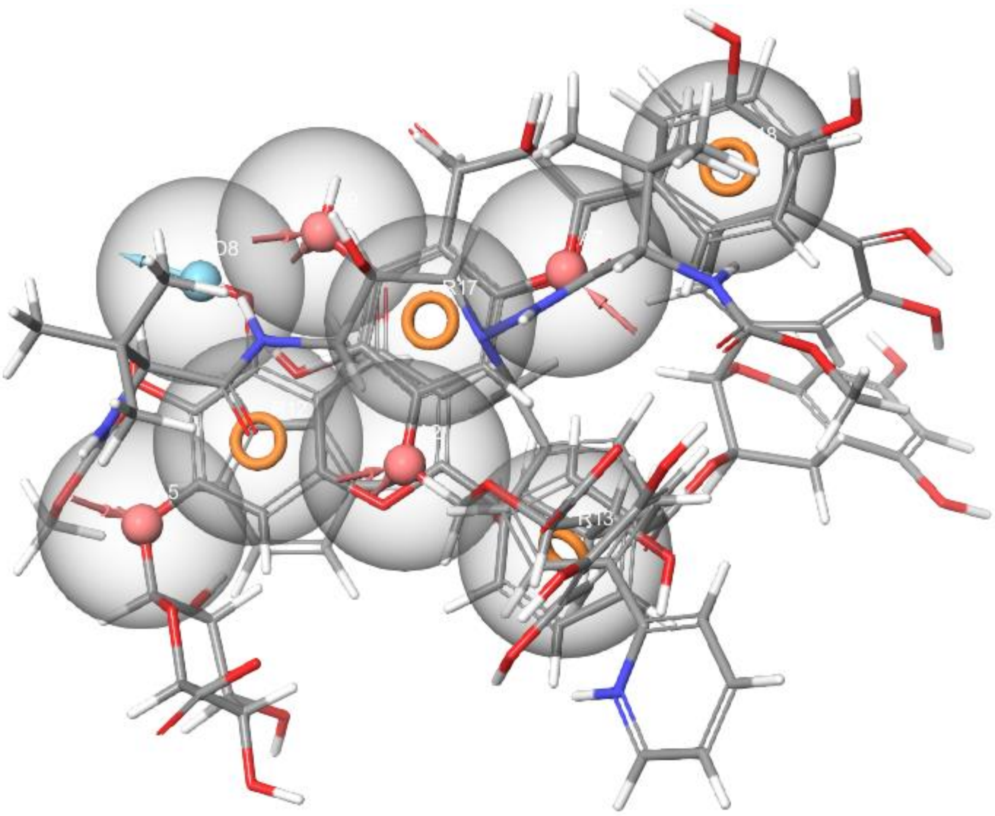

Figure S7. Pharmacophore model of the RBD site *c* of spike protein generated by the Multiple ligands method.

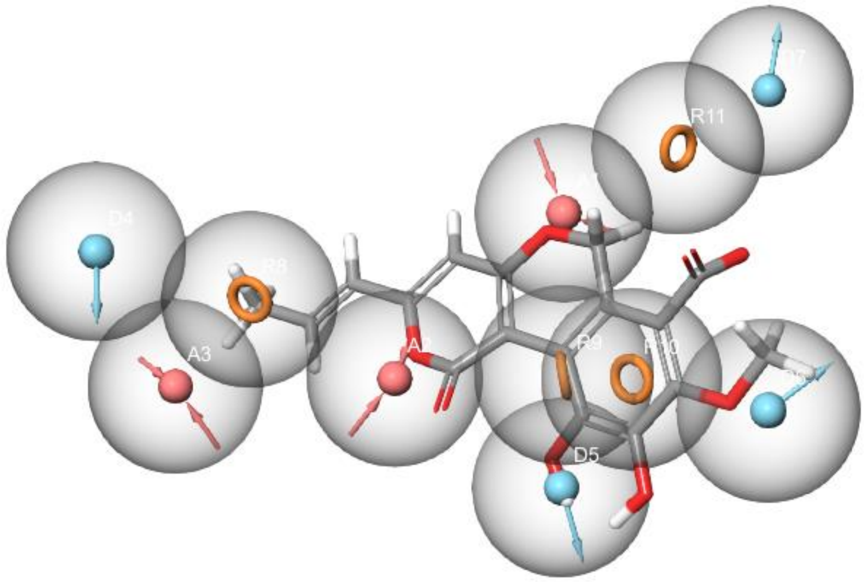

Figure S8. Pharmacophore model of the RBD site *c* of spike protein generated by the Merged hypothesis method.

Binding pocket: *d*

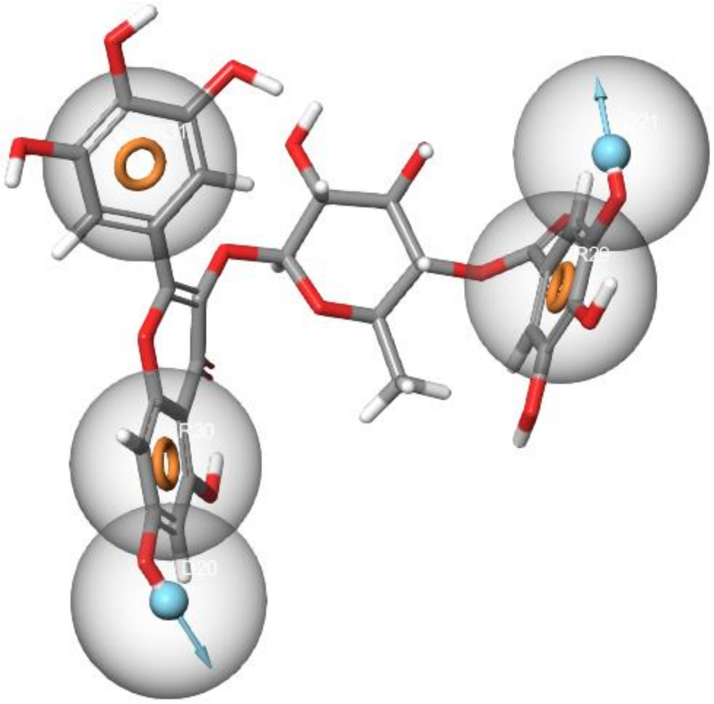

Figure S9. Pharmacophore model of the RBD site *d* of spike protein generated by the Receptor-ligand method.

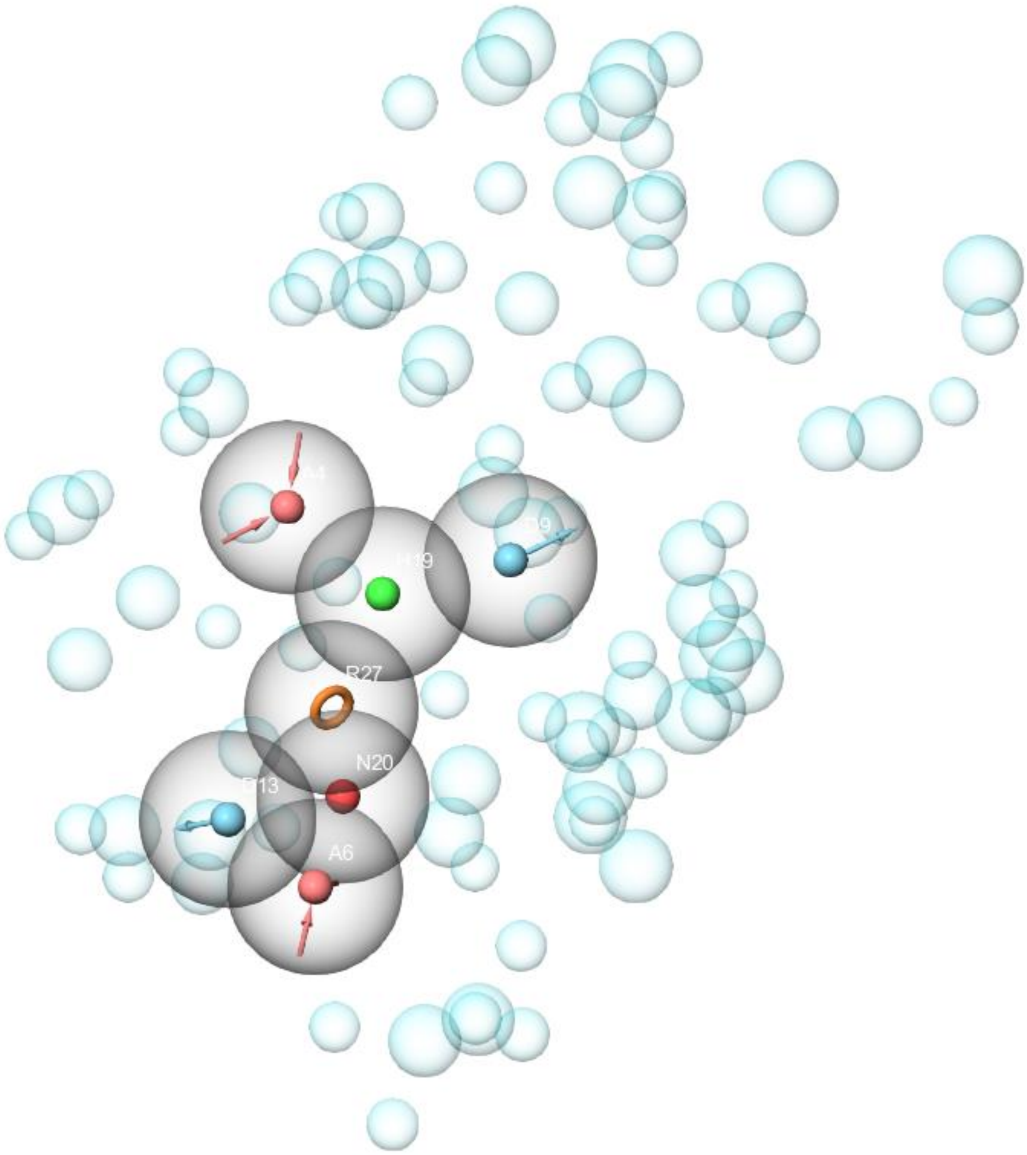

Figure S10. Pharmacophore model of the RBD site *d* of spike protein generated by the Receptor-cavity method.

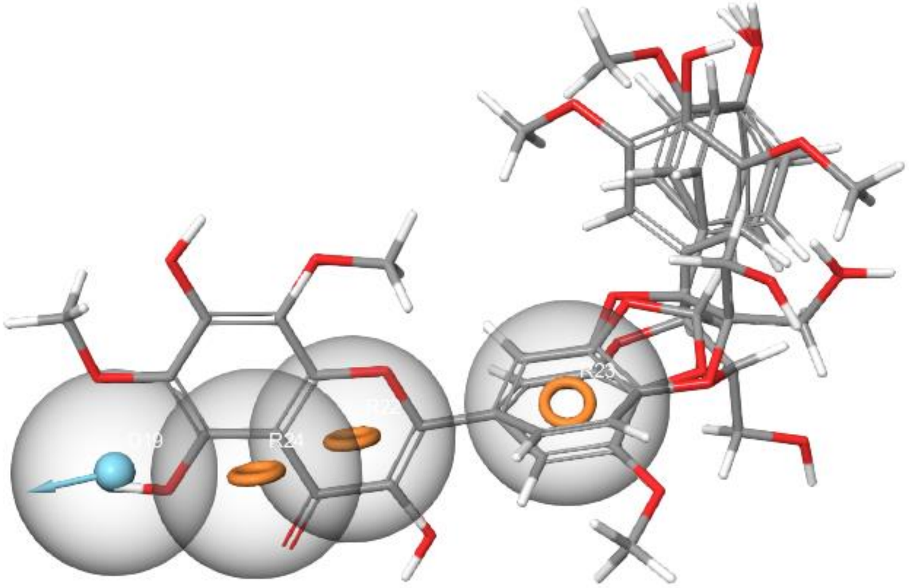

Figure S11. Pharmacophore model of the RBD site *d* of spike protein generated by the Multiple ligands method.

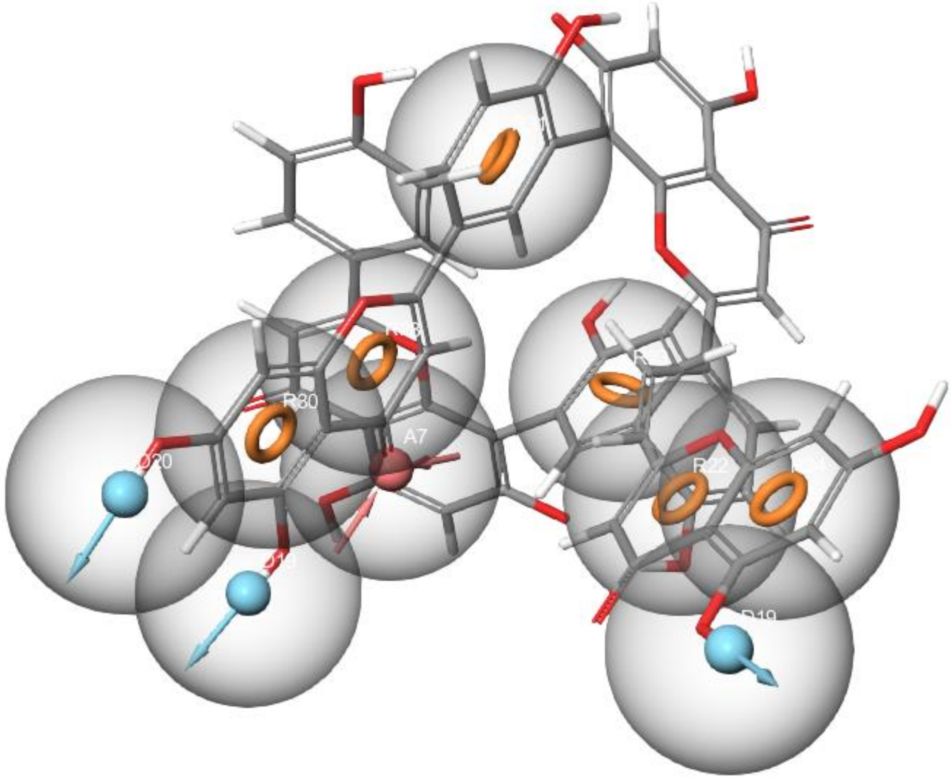

Figure S12. Pharmacophore model of the RBD site *d* of spike protein generated by the Merged hypothesis method.

Binding pocket: *e*

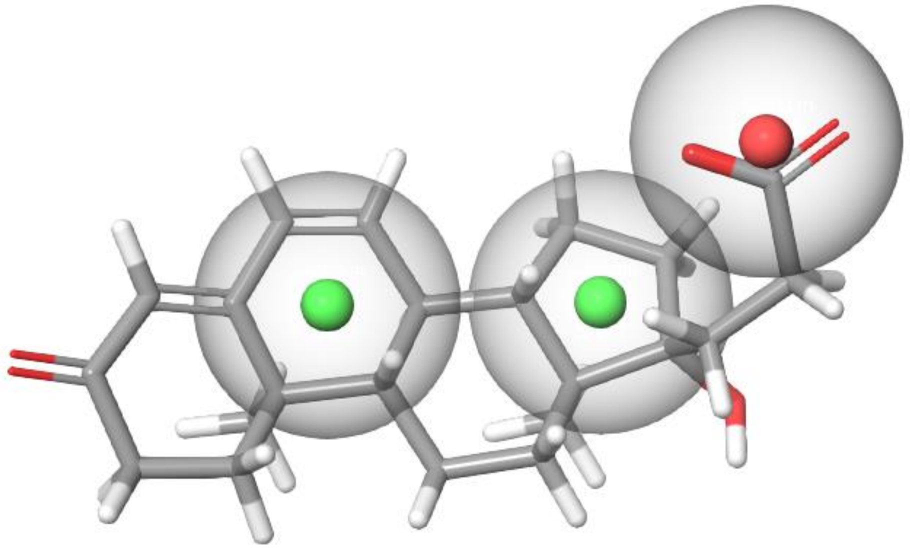

Figure S13. Pharmacophore model of the RBD site *e* of spike protein generated by the Receptor-ligand method.

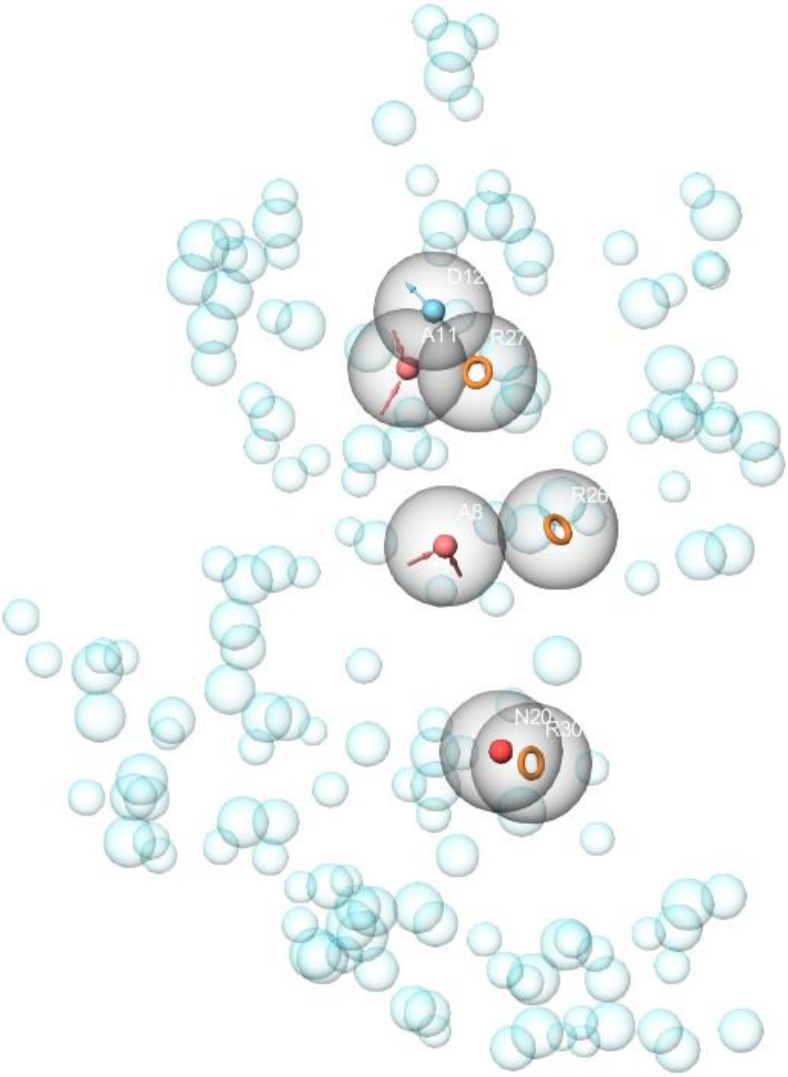

Figure S14. Pharmacophore model of the RBD site *e* of spike protein generated by the Receptor-cavity method.

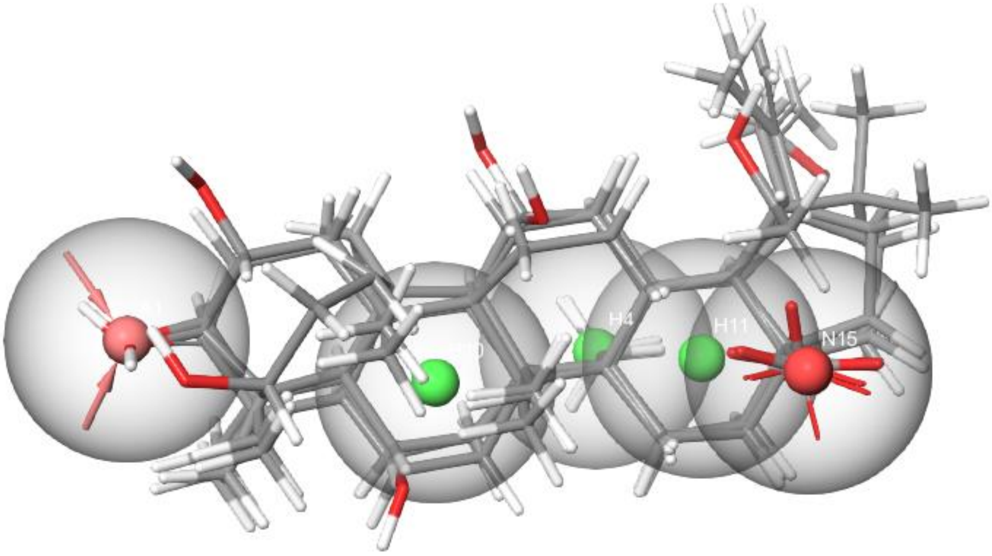

Figure S15. Pharmacophore model of the RBD site *e* of spike protein generated by the Multiple ligands method.

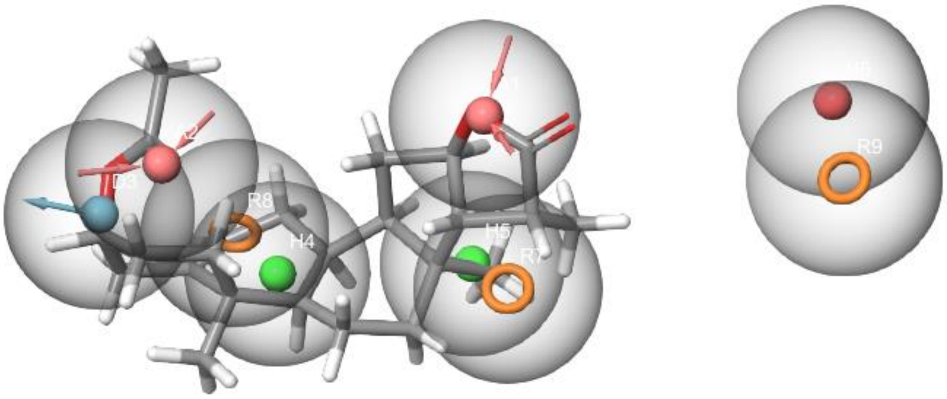

Figure S16. Pharmacophore model of the RBD site *e* of spike protein generated by the Merged hypothesis method.

Binding pocket: *f*

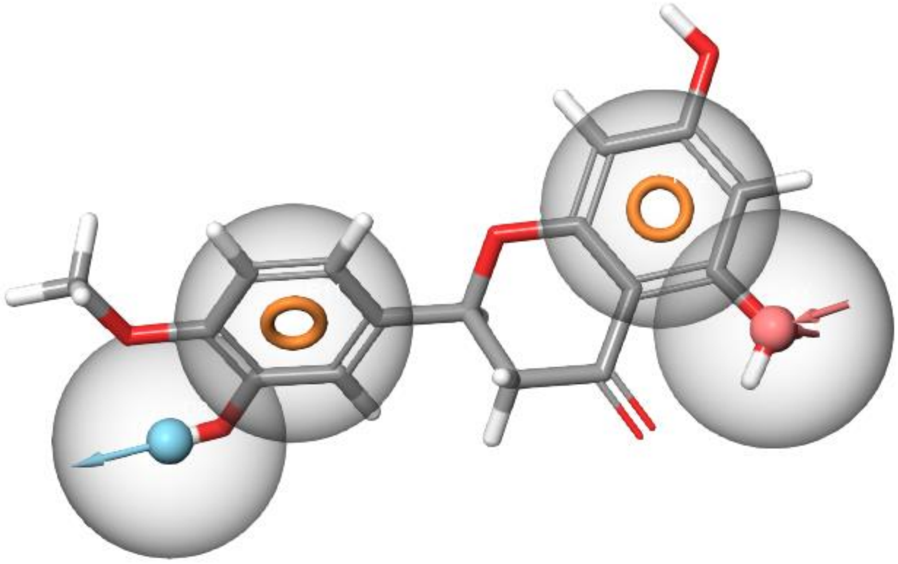

Figure S17. Pharmacophore model of the RBD site *f* of spike protein generated by the Receptor-ligand method.

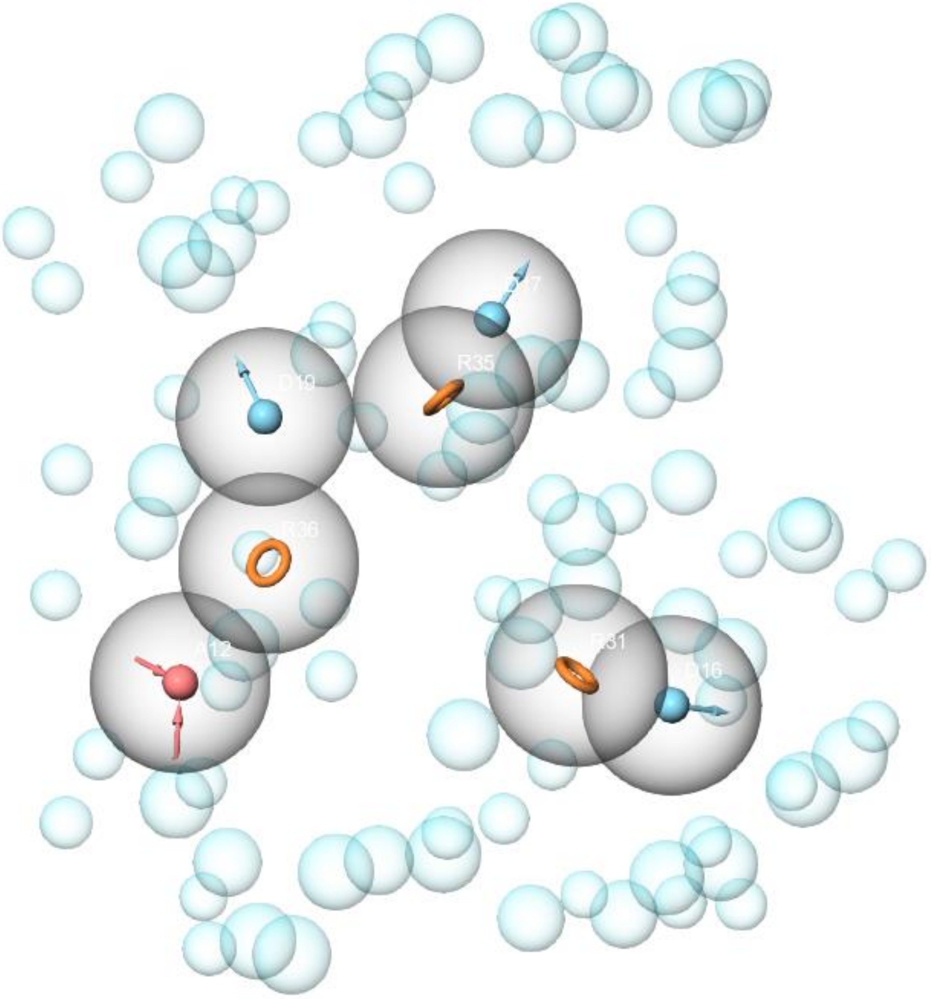

Figure S18. Pharmacophore model of the RBD site *f* of spike protein generated by the Receptor-cavity method.

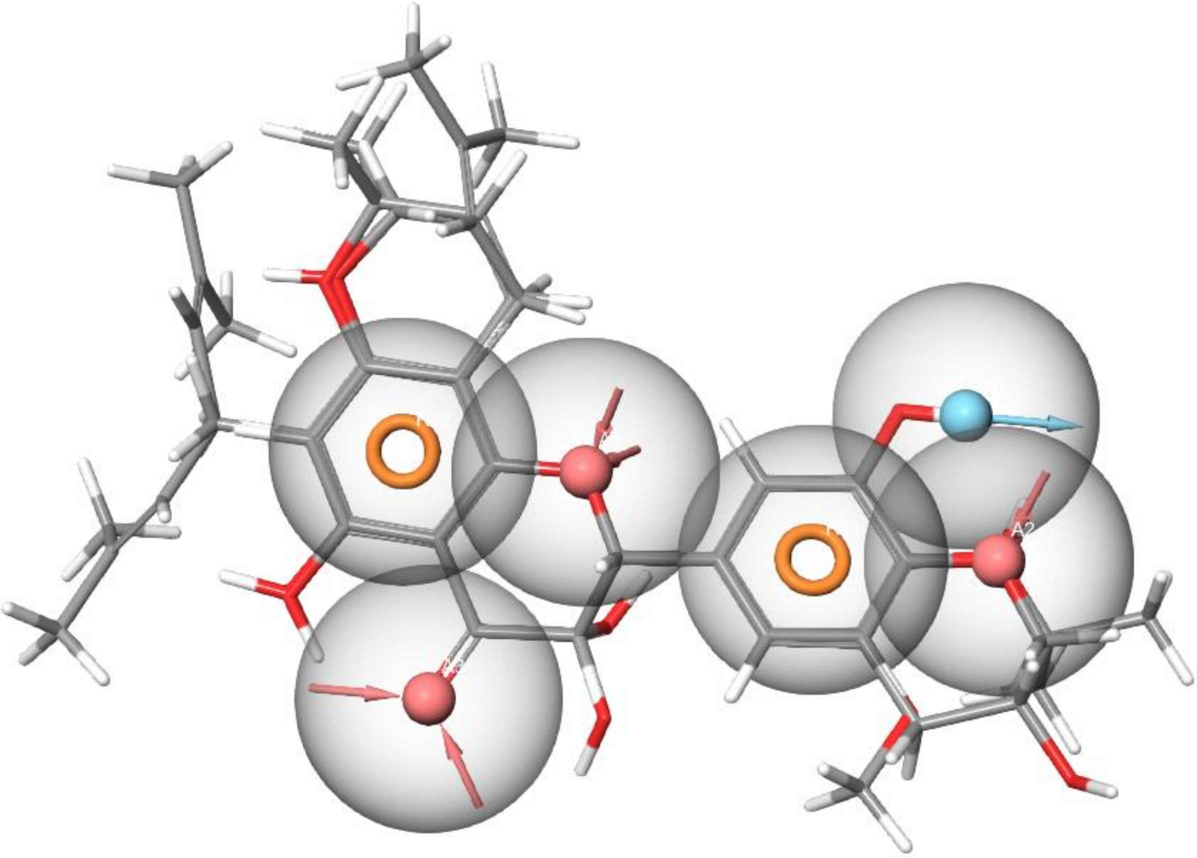

Figure S19. Pharmacophore model of the RBD site *f* of spike protein generated by the Multiple ligands method.

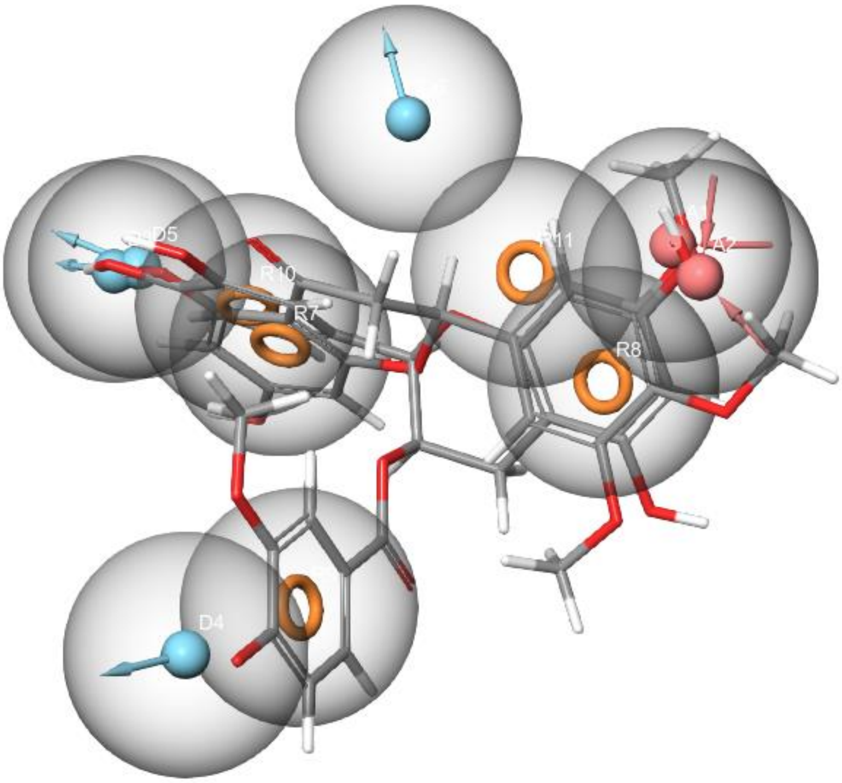

Figure S20. Pharmacophore model of the RBD site *f* of spike protein generated by the Merged hypothesis method.

Binding pocket: *g*

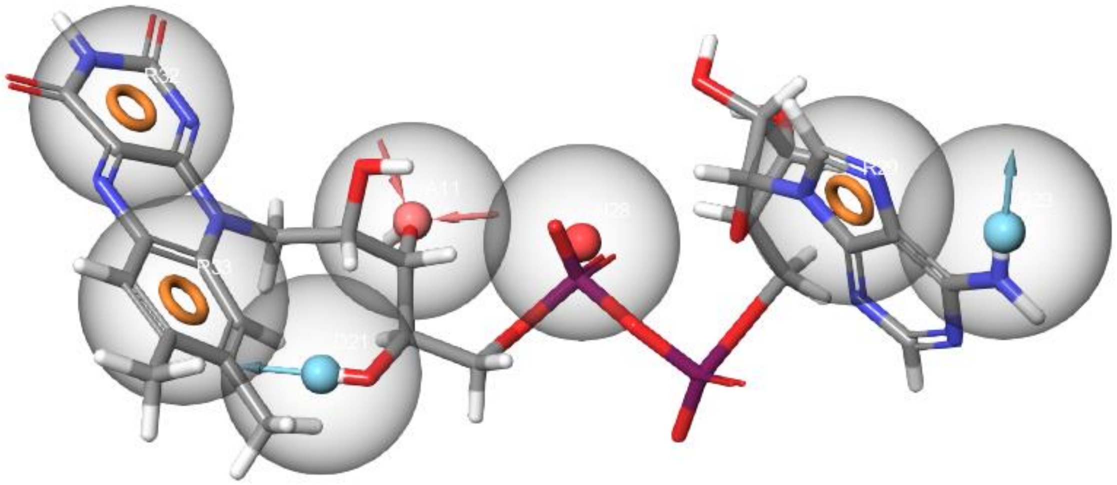

Figure S21. Pharmacophore model of the RBD site *g* of spike protein generated by the Receptor-ligand method.

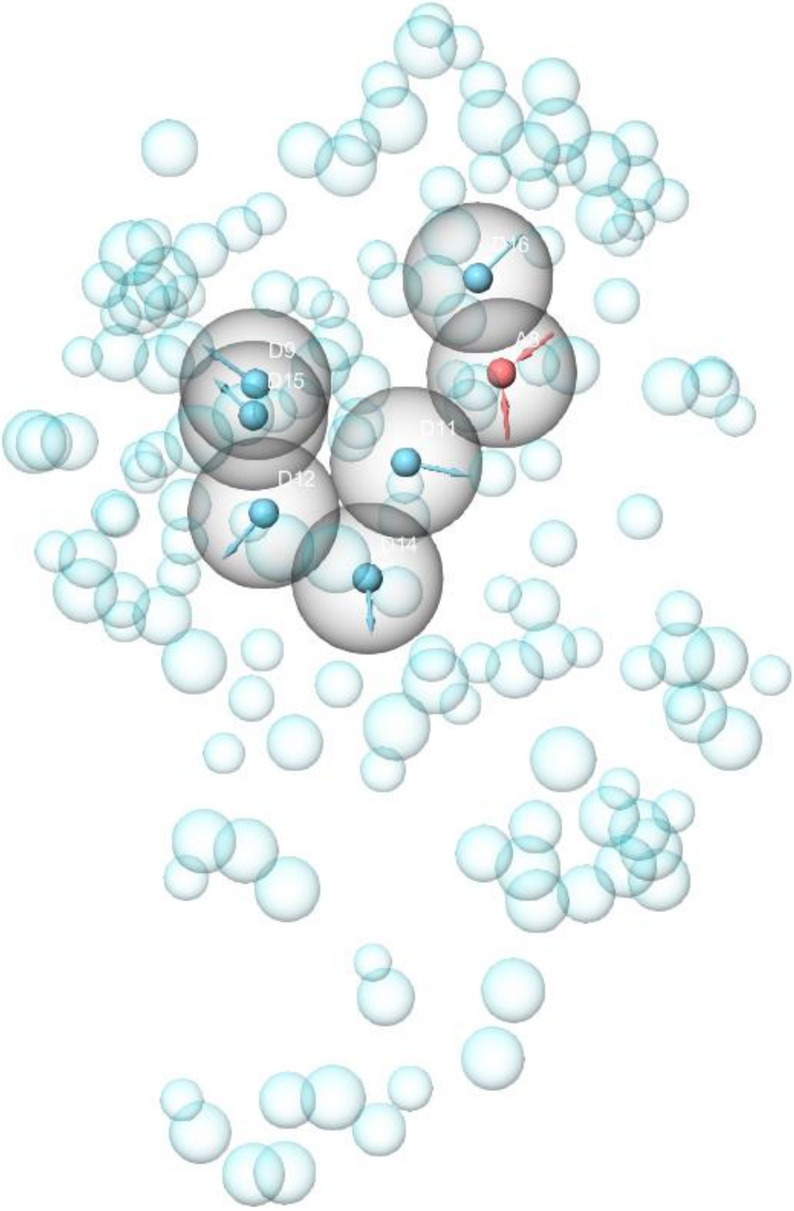

Figure S22. Pharmacophore model of the RBD site *g* of spike protein generated by the Receptor-cavity method.

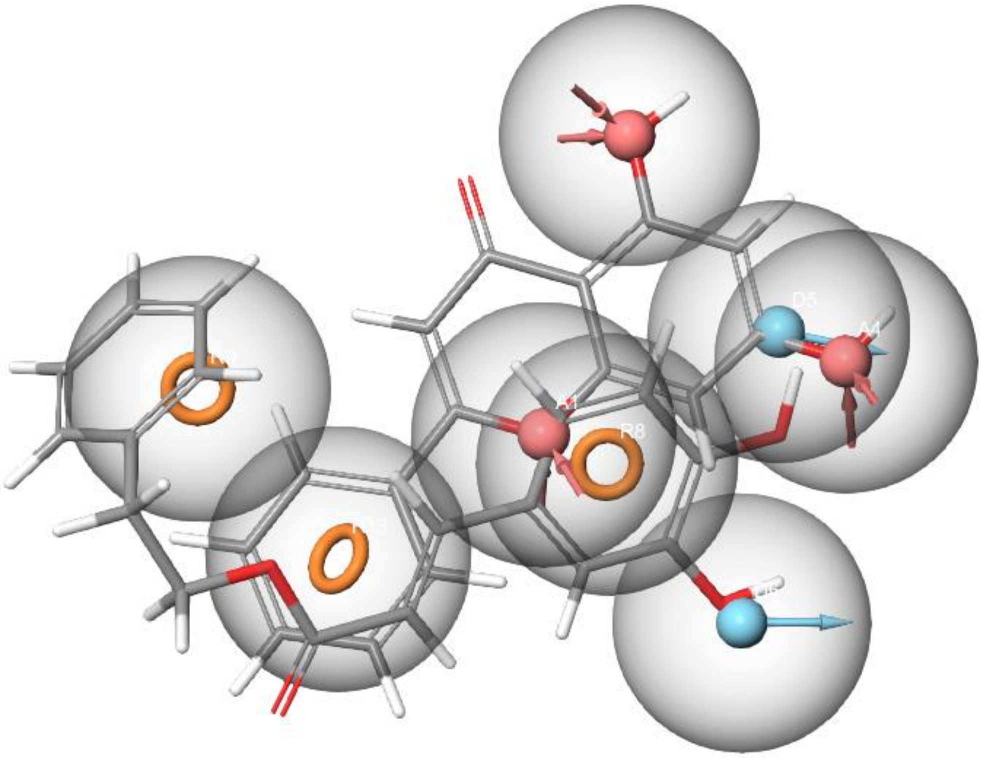

Figure S23. Pharmacophore model of the RBD site *g* of spike protein generated by the Multiple ligands method.

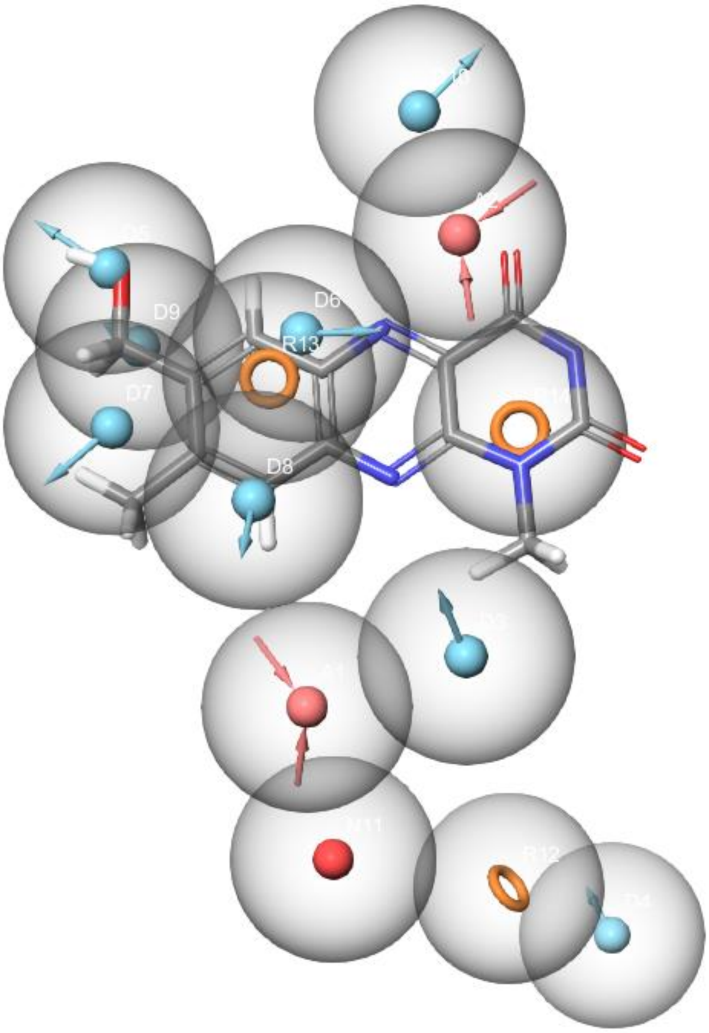

Figure S24. Pharmacophore model of the RBD site *g* of spike protein generated by the Merged hypothesis method.
